# Early-life proteomic and microbiome features signal obesity risk across 26 years of follow-up

**DOI:** 10.1128/msystems.01424-25

**Published:** 2026-05-28

**Authors:** Angelica P. Ahrens, Raquel Dias, Tuulia Hyötyläinen, Pär Anderson White, Matej Orešič, Eric W. Triplett, Johnny Ludvigsson

**Affiliations:** 1 Department of Microbiology and Cell Science, Institute of Food and Agricultural Sciences, College of Agricultural and Life Sciences, University of Florida 3463, Gainesville, Florida, USA; 2 School of Science and Technology, Örebro University 6233, Örebro, Sweden; 3 Crown Princess Victoria Children’s Hospital and Division of Pediatrics, Department of Biomedical and Clinical Sciences, Linköping University 4566, Linköping, Sweden; 4 School of Medical Sciences, Faculty of Medicine and Health, Örebro University 6233, Örebro, Sweden; 5 Turku Bioscience Centre, University of Turku and Åbo Akademi University 8058, Turku, Finland; 6 Department of Life Technologies, University of Turku 8058, Turku, Finland; Wageningen University, Wageningen, the Netherlands

**Keywords:** microbiome, metabolome, environmental toxins, inflammation, pregnancy, metabolic disease, diabetes, machine learning, ANGPTL4, FGF19, SULT2A1, bile acids, carbohydrates

## Abstract

Childhood obesity is rising globally. Yet, few studies have examined the microbiome and proteome in early childhood in relation to this outcome, and most are cross-sectional by design. Early-life factors in the ABIS birth cohort (*n* = 16,683) were associated with obesity up to age 26 (mean follow-up 25.3 years, range 23.7–26.5 years): psychosocial stressors, smoking, infections, and diet in the first year. We assessed biomarkers, including cord blood metabolome (*n* = 290) and proteome (*n* = 358), by liquid chromatography, mass spectrometry, and Olink. Gut microbial composition at age one (*n* = 1,743) was assessed using stool samples and 16S rRNA sequencing. In this prospective longitudinal cohort study, significant differences were found in infants with future obesity, including elevated angiopoietin-like 4 (ANGPTL4), follistatin, and hepatocyte growth factor (independently of maternal weight) and reduced isocaproic acid, tryptophan, and oleic acid, with prenatal mediation. *Akkermansia*, asaccharolytic bacteria (*Phascolarctobacterium* and *Senegalimassiliensia*), and equol-producers (*Adlercreutzia* and *Slackia*) were depleted. Machine learning models selecting 40 most predictive features showed long-term prediction from birth proteomics and bacterial taxa at age one (area under the curve [AUC] = 0.83 ± .05, *n* = 1,877) and additional metrics, for example, parental and child body mass index in the first 8 years (AUC = 0.89 ± .02, *n* = 1,877), suggesting durable biological encoding. Proteomic markers across folds included fibroblast growth factor 19, ANGPTL4, sulfotransferase family 2A member 1, and interleukin 20. These findings suggest clinically relevant biomarkers indicating early-life regulation of bile acid metabolism, lipid storage vs. oxidation, and immune–metabolic signaling and pathways to prospectively prevent childhood- and adult-onset obesity across a 26-year predictive gap.

**IMPORTANCE:**

Understanding the origins of obesity is critical for developing preventive strategies, and early life represents a particularly sensitive window. This study leverages a large, general-population cohort with prospectively collected data, including parental body mass index (BMI), cord blood proteomics, and the gut microbiome at age one, linked to obesity outcomes over 26 years. Using integrated machine learning models, we show that in addition to parental BMI, specific proteomic and microbial markers present in infancy can predict long-term obesity risk, highlighting the role of early metabolic programming. Several key markers point to bile acid signaling as a mechanism connecting early microbiome development with fat accumulation and insulin regulation. By identifying these early-life predictors long before obesity manifests, these results provide new insights into intergenerational risk and suggest measurable targets for preventing obesity and related metabolic disorders from the earliest stages of life.

## INTRODUCTION

Childhood obesity is rising globally (1), driven by biological, social, and environmental factors. Although diet and lifestyle contribute significantly, these alone are not sufficient. Several conceptual models of ecological influences have led to proposed interventions (2). Factors such as reduced breastfeeding, sedentary behavior, home environment, and infections associated with weight gain in children have been studied in recent meta-analyses (3–13). Early-life programming appears to be key to developing evidence-based, personalized treatment approaches (3) to reduce the health burden caused by obesity.

The intrauterine metabolic environment shapes the early microbiome, though its long-term impact on metabolism and obesity risk remains unclear. The link between parental and child obesity is well established (4), particularly during early childhood (5). However, multi-omic, prospective investigations are needed to understand the interactions of factors involved in obesity (6). The gut microbiota are a key mediator of host metabolism, facilitating monosaccharide absorption and hepatic lipogenesis (7). Certain species, like *Akkermansia muciniphila*, are consistently less abundant in individuals with obesity (8), while others like Firmicutes are more abundant. In children with obesity, altered levels of indole—produced by gut bacteria during tryptophan fermentation—suggest dysregulation of tryptophan metabolism (9). While probiotic interventions more broadly have shown variable success as treatments (10, 11), dietary prebiotics have shown moderate success in reducing body weight and improving metabolic profiles (12). Gut dysbiosis is a trigger that can cause inflammation, in part due to increased intestinal permeability (13).

Given that the first 1,000 days of life are critical for microbiome development, investigating early life processes is especially important (14, 15). The host–microbe–environment interactions are especially vulnerable to undernutrition, with intergenerational effects (14). Despite growing interest, few studies have examined the microbiome and proteome in early childhood, especially in the first years of life, in relation to future obesity. Most studies have been cross-sectional (16), with only five of the 42 focused on neonates or infants. A small study of forty 4- to 5-year-old children found increased *Enterobacteriaceae* and decreased *Akkermansia muciniphila*-like bacteria in children with weight issues. However, these differences coincided with obesity rather than preceding it, limiting causal interpretation (17). Another study of 2-year-olds provides preliminary evidence of an association, in which microbial differences preceded later weight problems at age 12, but its small sample size and lack of long-term follow-up into adulthood constrain its implications (18).

**Fig 1:**
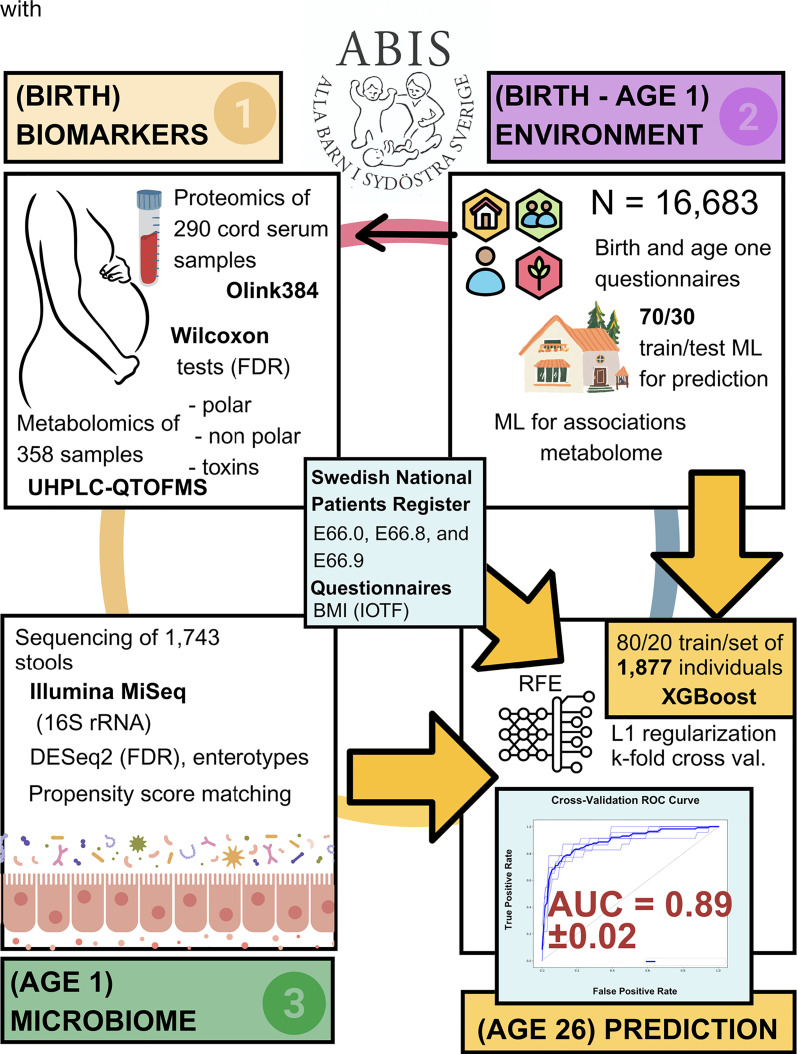
Study design. This cohort study is based on the All Babies in Southeast Sweden (ABIS) general population study (*N* = 16,683). Biomarkers were assessed for association with future obesity outcomes, including body mass index (BMI) classifications based on International Obesity Task Force (IOTF) standards and clinical diagnoses (ICD-10 codes E66.0, E66.8, and E66.9) through age 26. Biomarkers included metabolomics and targeted proteomics from cord blood, as well as microbiome sequencing from stool samples collected at approximately 1 year of age. Wilcoxon tests and differential expression analysis from binomial distribution modeling (via DESeq2) identified top metabolites, proteins, and taxa associated with obesity outcomes. Environmental and lifestyle exposures from pregnancy to the first year were modeled using machine learning (ML) with a 70/30 train-test split. The final integrated data set included 1,877 individuals with complete biological samples, which were used in the XGBoost ML pipeline. Recursive feature elimination (RFE) was employed for dimension reduction, and L1 regularization with k-fold cross-validation was applied to predict obesity diagnosis up to age 26. More details are provided in Materials and Methods. The final model achieved the area under the curve (AUC) of 0.89, using the top 40 features (taxa, proteins, and early-life risk factors) selected by RFE.

Here, in the All Babies in Southeast Sweden (ABIS) prospective longitudinal cohort study, we examined the cord blood proteome and gut microbiome at age one year in relation to obesity outcomes over 26 years of follow-up. This study of a general population enables the prospective identification of early predictors of long-term obesity risk (Fig. 1). Understanding how the intrauterine metabolic environment and early microbial development contribute to the intergenerational transmission of obesity risk could guide preventive strategies, early-life interventions, and development of potential biomarkers.

## MATERIALS AND METHODS

### Study design

The ABIS study (19–21) is a prospective, general population-based cohort (19) of children born in southeast Sweden from October 1997 to 1999. Parents were recruited from nine obstetric clinics across Östergötland, Småland, Blekinge, and Öland, with 17,055 out of 21,700 (78.6%) families providing informed consent, of whom 16,683 had answered the birth questionnaires. The ABIS study shows a balanced distribution of biological sex, with 48.2% female, and as it consists only of children born in Sweden (primarily to Swedish parents), sociocultural constructs like race and ethnicity were not collected. The ABIS register was connected to the Swedish National Patient Register (NPR) (22), giving the confirmed clinical diagnosis of obesity (23).

### Definitions of obesity cases and controls and analytical framework

Obesity was defined using two complementary definitions. First, BMI was calculated from parent-reported height and weight from the 3-, 5-, 8-, 10-, and 19-year questionnaires, with weight classifications following IOTF (24) standards. Second, registry-based clinically diagnosed obesity was identified (*n* = 407 of 16,683) using International Classification of Diseases (ICD-10) codes E66.0, E66.8, and E66.9, as recorded in participants’ medical records, via the Swedish National Patient Register through December 2023. Controls were defined as normal weight by IOTF standards, based on a parent-completed questionnaire, and with no future autoimmune or neurodevelopmental diagnosis through December 2023.

Questionnaire- and BMI-based overweight and obesity classifications derived from parent-reported height and weight, as well as perinatal and prenatal features, were used to stratify or group participants in the proteomic and microbiome analyses and as predictors in single-layer machine learning analyses linking features to metabolomic data. These measures were not used as outcomes in the integrated multi-omic machine learning models, which focused exclusively on clinically diagnosed obesity obtained from the Swedish National Patient Register (ICD-10 codes), thereby avoiding reliance on self-reported anthropometric measurements for predictive modeling. Given known measurement error in parent-reported anthropometrics, age- and sex-specific IOTF obesity categories were used to improve robustness and reduce sensitivity to modest reporting inaccuracies.

The primary clinical endpoint for etiologic inference and predictive modeling was the registry-confirmed obesity diagnosis obtained from the Swedish NPR (22). While this outcome underestimates population prevalence, it represents a highly specific and physician-recorded diagnosis, providing greater confidence in outcome validity.

### Statistics

#### Associations between questionnaire data (birth and 1-year surveys) and future obesity outcomes

For exploratory associations between early-life questionnaire variables and future obesity, odds ratios (ORs) were calculated from 2 × 2 contingency tables in SPSS for exposures including gestational diabetes, parental education, parental BMI, infections, and antibiotic use. Odds ratios represent unadjusted associations between exposure and outcome. These analyses were conducted using both obesity definitions: age- and sex-specific IOTF classifications derived from parent-reported questionnaires and registry-confirmed clinical obesity diagnoses.

For this investigation, we primarily focused on the prenatal/birth and 1-year questionnaire environmental data, as these exposures precede the development of obesity and minimize potential reverse causation. Gestational diabetes of the mother was additionally assessed using Swedish National Patient Register data (ICD-10 code O24.4).

In addition to early-life factors collected at birth and 1 year of age, parental BMI was also analyzed in two distinct contexts. First, to characterize familial patterns of adiposity across early childhood, parental BMI measurements obtained from follow-up questionnaires (up to age eight of the child) were used in correlation analyses to examine associations with child BMI at multiple ages. Second, parental BMI was included as a predictor of clinically diagnosed obesity through adolescence and early adulthood, where it was treated as a stable background characteristic reflecting long-term familial risk rather than a child-level exposure.

Given the large number of interrelated early-life exposures and the descriptive aim of this component of the study, multivariable adjustment was not applied. These analyses were intended to identify early-life patterns associated with later obesity risk and should not be interpreted as estimates of independent causal effects.

#### Metabolomic analysis of cord serum and associations with obesity (by IOTF classification)

Cord serum was analyzed in 358 ABIS children, as described previously (19). Briefly, lipidomics profiling was carried out using an ultra-high-performance liquid chromatography quadrupole time-of-flight mass spectrometry (UHPLC-QTOFMS from Agilent Technologies; Santa Clara, CA, USA) and analysis with an ACQUITY UPLC BEH C18 column (2.1 mm × 100 mm, particle size 1.7 μm) by Waters (Milford, MA, USA). Polar and semipolar metabolites were analyzed using an Acquity UPLC system coupled to a triple quadrupole mass spectrometer (Waters Corporation, Milford, MA, USA) with an atmospheric electrospray interface operating in negative-ion mode, with quantification of bile acids (BAs) and PFAS using a 7-point internal calibration. Data were processed using MZmine 2.53. Pooled quality control samples were included, along with a reference and compound standards, and extracted blank samples. Polar metabolites were assessed against BMI IOTF classifications at age 5 years using the Kruskal–Wallis test.

#### Predicting metabolites and exogenous compounds in cord blood from prenatal/perinatal features

To predict metabolite and exogenous toxin concentrations from prenatal and perinatal factors, and vice versa, we employed ML models including Extreme Gradient Boosting (XGBoost), Support Vector Machines (SVM), Extra Trees, Random Forest (RF), and Logistic Regression (LR), using normalized metabolomic data as a continuous variable. The goal was to assess which early-life factors contain predictive information about the composition of the cord blood. Model performance was assessed using AUC and F1 score. To interpret the direction of the effect of each predictor, explainable AI via SHapley Additive exPlanations (SHAP) was used to learn the contribution of features in the predictions. These associations serve to identify predictive patterns rather than establish causal effects.

#### Proteomic analysis of cord serum

Cord serum samples were processed by Olink (Uppsala, Sweden), using Olink’s Explore 384 Inflammation 1 and 2 panels and the Target Immune Response panel. Cases were intentionally oversampled to overcome class imbalance. Internal and external controls were run for batch effects and technical variations. Normalized protein expression (NPX) values were calculated as a relative protein quantification unit on a log2 scale, and data intensity was normalized.

Initial differences between case/control were assessed using Wilcoxon test statistics comparing NPX values, based on BMI classifications across time (self-reported questionnaires) or on clinical diagnosis from the Swedish National Patient Register (up to age 26). Median values for each group and the effect size (Cohen’s *d*) between the two groups were calculated. Comparisons included the following: 2–10 years global comparison (218 controls vs. 72 a BMI corresponding to obesity at least once across the timepoints) and BMI at 2 (129 controls vs. 45 overweight/obese), 5 (92 controls vs. 51 overweight/obese), and 8 (43 controls vs. 19 overweight/obese) years. For the analysis based on clinical diagnosis, 204 controls and 84 future obesity cases were compared, with E66.0, E66.8, and E66.9 as the qualifying ICD-10 codes.

#### Sequencing of stool samples at 1 year of age

A stool sample was collected from the participating child’s diaper, at a mean age of 11.93 ± 2.94 months, using a sterile spatula and collection tubes provided by the WellBaby Clinic, as previously described (19). Briefly, samples were immediately frozen after collection, either at the infant’s home or at the clinic. For home collections, freeze clamps were used to maintain frozen conditions during transport to the clinic, where samples were subsequently stored at −80°C.

DNA extraction and sequencing of 16S rRNA gene amplicons from 1,748 stool samples were done as previously described (19). Briefly, a total of 1,748 samples were sequenced in 10 pools using Illumina MiSeq 2 × 300 bp at the Interdisciplinary Center for Biotechnology Research (ICBR) at the University of Florida, Gainesville, Florida, USA, following established protocols. Amplicons for targeted V3-V4 16S rRNA sequencing were produced using Standard Illumina Read 1 sequencing/indexing primers 341F (NNNNCCTACGGGAGGCAGCAG) and 806R (GGGGACTACVSGGGTATCTAAT). Forward primer was as follows: 5′-P5-adapter-linker-SBS3-16S-3′, 5′-AATGATACGGCGACCACCGAGCIWTHTAYGGIAARGGIGGGIATHGGIAA-3′. Reverse primer was as follows: 5′-P7 adapter-linker-barcode-SBS12-16S-3′, 5′-CAAGCAGAAGACGGCATACGAGAT-(barcode)-GTGACTGGAGTTCAGACGTGTGCTCTTCCGSTCTGGGGACTACVSGGGTATCTAAT-3′. For pooling, barcodes 11 nucleotides in length were used. Each PCR sample was spin-column purified and quantified by Qubit prior to pooling.

Paired ends were joined and demultiplexed in QIIME1, sample inference done using DADA2, and taxonomy assigned using the SILVA database, discarding five samples with low read counts. The resulting pool comprised 107,959,956 reads (with a minimum of 10,059 and a maximum of 776,158 reads per sample), including an average of 61,939.1 reads per sample (57,844 median) and 13,197 unique amplicon sequence variants (ASVs), representing 511 genera and 931 species.

#### Differential gut microbiome analysis

Differential taxonomic analysis was conducted using a negative binomial generalized linear model approach (DESeq2 [25]) with FDR correction. Significant taxa were defined as those with false discovery rate (FDR)-adjusted *P* < 0.05 and normalized base mean counts of abundance ≥10, representative of the average of the normalized counts (adjusted for sequencing depth across samples) for the taxon across all groups in that data set. This is calculated as the geometric mean of the counts across all samples, regardless of outcome.

A total of 598 controls and 50 future clinical obesity cases, with a qualifying diagnosis of E66.0, E66.8, or E66.9, were included in the analysis. Differential abundance was tested using DESeq2. Independent filtering of features with very low mean counts was applied automatically by DESeq2 to optimize detection power. No manual pre-filtering of taxa was performed.

For the IOTF-based comparisons using the matchIt R package, a propensity score matching of a 3:1 ratio of controls to cases addressed factors related to prenatal, birth, and early diet influences: biological sex, parental education level at birth, maternal smoking during pregnancy, gestational age at birth, mode of delivery, breastfeeding duration, mother’s BMI, gestational diabetes, and antibiotic exposure. The matching method used was nearest neighbor. The resulting data sets included 13 children who were obese by age 3 against 39 controls of normal weight (400 taxa represented), and 22 children with obesity by age 5 against 66 controls (464 taxa represented). As before, independent filtering was applied using DESeq2 defaults, and no additional, manual pre-filtering was performed.

#### Integrated, multi-omic machine learning to predict future obesity diagnosis

ML models were developed to predict future clinically diagnosed obesity using integrated molecular features (microbiome and proteomics) from 1,877 ABIS individuals, where either microbiome or proteomic data were available, including 114 with a clinical obesity diagnosis from the Swedish National Patient Register. For the ML prediction models, XGBoost, a gradient-boosted decision tree algorithm, was implemented in Python, with the outcome variable encoded as a binary classification (0 = no obesity diagnosis; 1 = clinical obesity diagnosis). The XGBoost models were implemented using the XGBClassifier with the following hyperparameters: n_estimators = 300, max_depth = 4, learning_rate = 0.05, alpha = 1 (L1 regularization to induce sparsity), eval_metric = “logloss,” random_state = 42, and scale_pos_weight set to the ratio of controls to cases within each training fold to account for class imbalance. The logloss evaluation metric and L1 regularization (alpha = 1) were used to induce sparsity. Default hyperparameters were used based on established performance in similar biomedical prediction tasks with imbalanced data. All ML models were implemented in Python 3.10.12 using: XGBoost (v3.1.3, dmlc implementation), scikit-learn (v1.8.0) for preprocessing and cross-validation, SHAP (v0.50.0) for model interpretation.

Initially, models were trained on the molecular features alone, and then with metadata incorporated, including the first-year questionnaire as well as parental and child BMI up to age 8 to evaluate incremental predictive value, while considering possible interactions between biological and non-biological features. A total of 683 features were brought into testing, including 230 bacterial and 363 proteomic, prior to RFE selection within each fold. Missing data were handled using scikit-learn’s SimpleImputer (median strategy) within the testing and training sets separately, and within each fold, to avoid data leakage.

For both approaches, the data set was split into 80% and 20% training and testing sets with a fixed random state for reproducibility. Model performance was evaluated using stratified fivefold cross-validation to preserve case–control proportions across folds. Within each fold, the model was trained on four-fifths of the data and evaluated on the held-out fold. No information from the test fold was used during feature selection or model fitting. To reduce dimensionality, feature selection was performed using RFE with an L1-regularized XGBoost estimator. Importantly, RFE was conducted within each training fold only to prevent information leakage. For each fold, the top 40 features were retained based on model-based feature importance rankings. We evaluated models using both 40 and 50 features selected via RFE. Performance was nearly identical, suggesting that the top 40 features capture the majority of the predictive signal. We selected 40 features for the final model to maximize interpretability while minimizing overfitting risk, given our 114 cases.

The number of times each selected feature was chosen was counted, so that the key features of obesity risk across folds could be identified. Predictive performance was assessed using the area under the receiver operating characteristic curve (AUC), precision, recall, and F1 score. Performance metrics were calculated independently for each fold and summarized as mean ± standard deviation across cross-validation folds. An average ROC curve was constructed by interpolation across a common false-positive rate grid. Consensus features were defined as those selected by RFE in at least three cross-validation folds, and a final XGBoost model was then trained on the full data set using only the consensus features, with class imbalance addressed via scale_pos_weight and L1 regularization (alpha = 1), to extract the relative importances.

This modeling framework was designed to minimize overfitting, ensure independence between training and testing data, and provide conservative estimates of predictive performance. The combination of within-fold feature selection, L1 regularization, and stratified cross-validation substantially reduced overfitting risk. Importantly, the integrated predictive models relied exclusively on registry-confirmed clinical obesity diagnoses as the primary outcome, thereby avoiding dependence on self-reported anthropometric measurements.

## RESULTS

**Fig 2:**
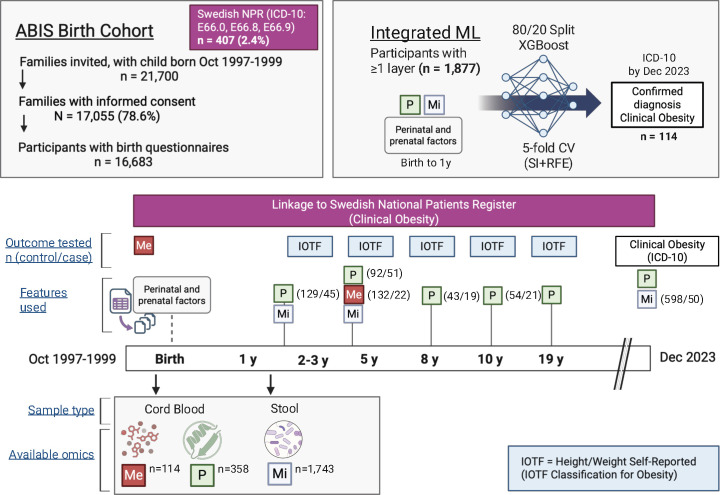
Analytical process. The pipeline for the investigation, demonstrating the metabolomic (Me), proteomic (P), and microbial (Mi) data sets used in each analysis.

An overview of data availability across questionnaire and omics-based analyses is provided in Fig. 2. BMI-based outcomes (defined according to standard IOTF classifications on the questionnaire-based data across time) were used to characterize growth patterns and stratify participants for proteomic and microbiome analyses, as well as to assess associations with metabolite levels in cord blood. They were not used as outcomes in the integrated multi-omic machine learning models, which relied exclusively on clinically diagnosed obesity from the National Patient Register.

A total of 407 ABIS children were diagnosed with obesity (BMI across time shown in Table 1). While only 12.4%–13.8% of children with clinically diagnosed obesity were obese at age 3, 27.8% of females with obesity at age 5 remained clinically obese, as did 53.7% of males (Table 1). Individuals who met IOTF obesity criteria but did not receive a clinical diagnosis were retained in BMI-based analyses but were classified as non-cases for registry-based analyses, reflecting known underdiagnosis of obesity in clinical care rather than exclusion or misclassification. Registry-based ICD-10 obesity diagnoses were used as the primary clinical outcome for proteomic, microbiome, and integrated multi-omic analyses.

**TABLE 1:** Obesity in the ABIS cohort^*a*^

Parameter	IOTF	Male future diagnosis of obesity				Female future diagnosis of obesity			
		No		Yes		No		Yes	
		Count	%	Count	%	Count	%	Count	%
BMI at 2 years	Normal or overweight	4,055	98.10	50	86.21	3,690	97.46	113	87.60
	Obese	78	1.89	8	13.79	96	2.54	16	12.40
BMI at 5 years	Normal or overweight	3,392	97.11	25	46.30	2,974	96.12	78	72.22
	Obese	101	2.89	29	53.70	120	3.88	30	27.78
BMI at 8 years	Normal or overweight	1,531	98.27	15	75.00	1,371	97.79	27	75.00
	Obese	27	1.73	*5*	25.00	31	2.21	9	25.00
BMI at 10 years	Normal or overweight	1,640	97.97	10	55.56	1,616	98.54	28	75.68
	Obese	34	2.03	8	44.44	24	1.46	9	24.32
Obese at 19 years	Normal or overweight	2,316	95.35	32	76.19	2,753	95.23	61	71.76
	Obese	113	4.65	10	23.81	138	4.77	24	28.24

^*a*^ Prevalence of obesity based on clinical diagnosis (ICD-10 codes) and IOTF-defined classifications from self-reported data at 3, 5, 8, 10, and 19 years.

### Associations with early-life infection, lifestyle, and environment

**Fig 3:**
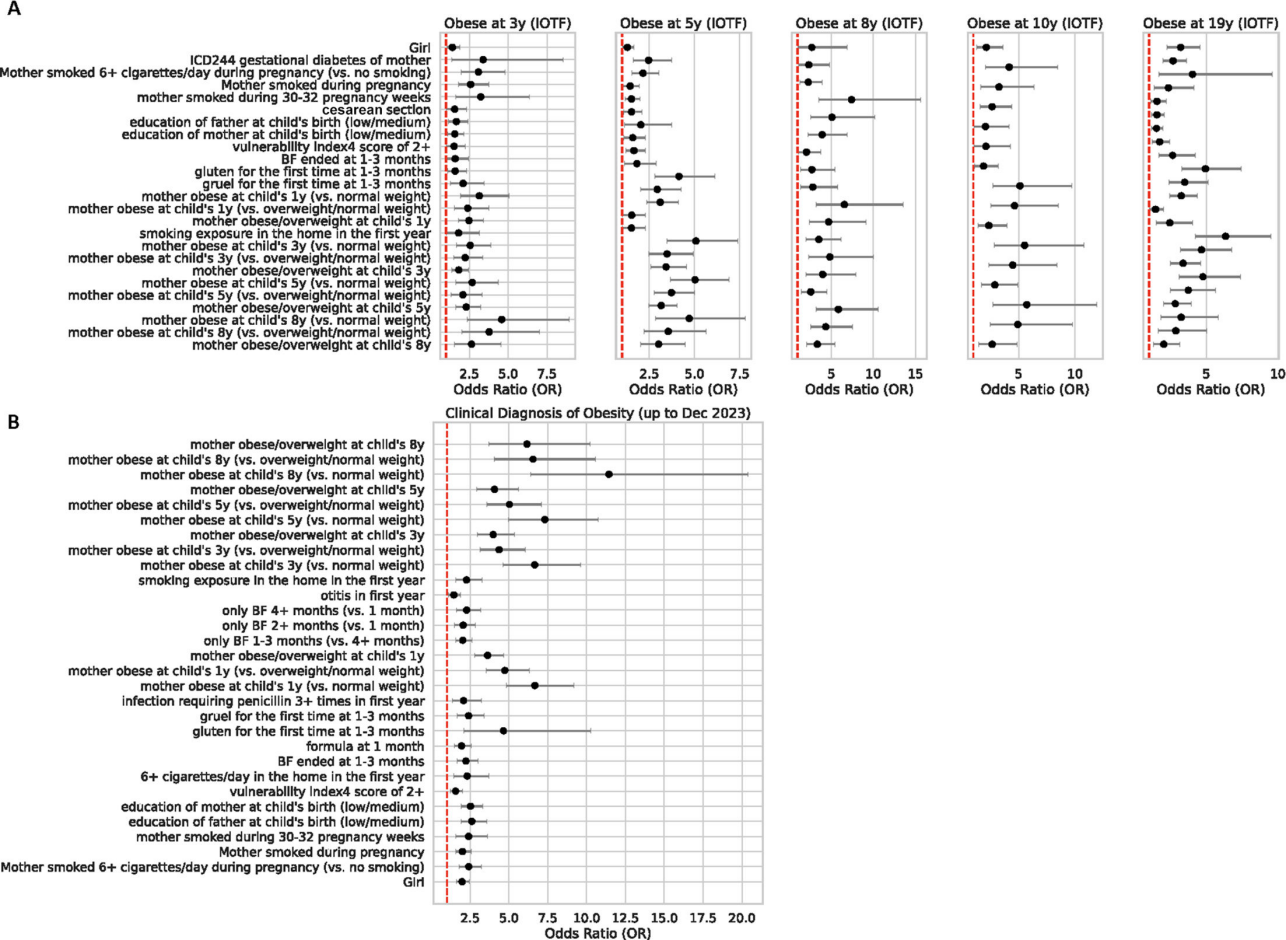
Early-life factors associated with future clinical obesity diagnosis or obesity classification according to IOTF standards (ages 3–19 years). ORs and 95% confidence intervals (CIs) are provided for each reference group and comparison, stratified by IOTF classifications and future obesity diagnosis (up to December 2023). Factors associated with (**A**) obesity classifications based on BMI at 3, 5, 8, 10, and 19 years of age against IOTF standards, and (**B**) clinical diagnosis of obesity from the medical record. ORs represent unadjusted associations between exposure and outcome.

Parent questionnaires (administered at birth and age one, *n* = 16,683) were assessed for associations with either clinical diagnosis of obesity or obesity classification based on IOTF standards (at 3, 5, 8, 10, and 19 years). Significant findings are presented in Fig. 3 (see also Fig. S1).

Females were more likely than males to later receive an obesity diagnosis (OR = 1.97 [1.61–2.43, 95% CI], *P* < 0.0001) or to be obese at ages 3 or 5 (OR = 1.41 [1.06–1.87, 95% CI], *P* < 0.019 and OR = 1.29 [1.02–1.64], *P* = 0.036, respectively). Maternal gestational diabetes mellitus (ICD-10 O24.4) was associated with obesity at age 3 (OR = 3.39 [1.35–8.53], *P* = 0.0096) only. Lower maternal and paternal education levels were also associated with a later clinical diagnosis of obesity in this child (OR = 2.52 [1.92–3.30], *P* < 0.0001 and OR = 2.61 [1.91–3.567, 95% CI], *P* < 0.0001, respectively), as well as with obesity measurements at ages 3, 5, 8, 10, and 19 years (ORs = 1.44–2.28). Children born to overweight or obese mothers had a 1.3- to 6.2-fold increased risk of obesity, depending on the classification and timepoint. Maternal obesity carried the greatest risk, increasing the likelihood of clinical obesity diagnosis in children by 3.1–11.4 times (all *P*’s < 0.0001). Delivery mode was not significantly associated with obesity risk, though cesarean section showed marginal associations at the 3- and 19-year time points. Higher psychosocial vulnerability index scores (19, 26) were associated with obesity at 3 and 5 years as well as clinical diagnosis of obesity later in life (OR = 1.57 [1.21–2.02, 95% CI], *P* = 0.0006).

Otitis media increased the odds of clinical obesity diagnosis (OR = 1.45 [1.11–1.90, 95% CI], *P* = 0.0062), while stomach flu was associated with obesity at age 10 (OR = 1.91 [1.14–3.22, 95% CI], *P* = 0.0143). Having frequent infections requiring penicillin (three or more times in the first year) increased the odds of a future obesity diagnosis by twofold (OR = 2.08 [1.32–3.22, 95% CI], *P* = 0.001). Maternal smoking during pregnancy (OR = 1.73–3.23) and exposure to smoking in the home during the first year also elevated risk (OR = 1.58–2.44). Some, but not many, dietary exposures in the first year were identified. Early cessation of breastfeeding (at 1–3 months) was associated with obesity at age 3 and 5, as well as later clinical obesity diagnosis. Early introduction of formula (at 1 month) and carbohydrate-heavy gruel (at 1–3 months) were significantly linked to obesity at all time points—particularly strong for clinical diagnosis (OR = 2.40 [1.68–3.42, 95% CI], *P* < 0.0001). Early gluten introduction (at 1–3 months) was a significant exposure (*P* = 0.0001).

Although obesity is multifactorial, an AUC of 0.66–0.67 was achieved using a model with a limited set of early lifestyle factors: early introduction of gruel (a carbohydrate-dense porridge, previously linked to high BMI at age 12 and 18 months (27), penicillin-treated infections during the first year, otitis, prenatal and household smoking, biological sex, and parental education at birth.

### Associations with proteomic markers at birth

**Fig 4:**
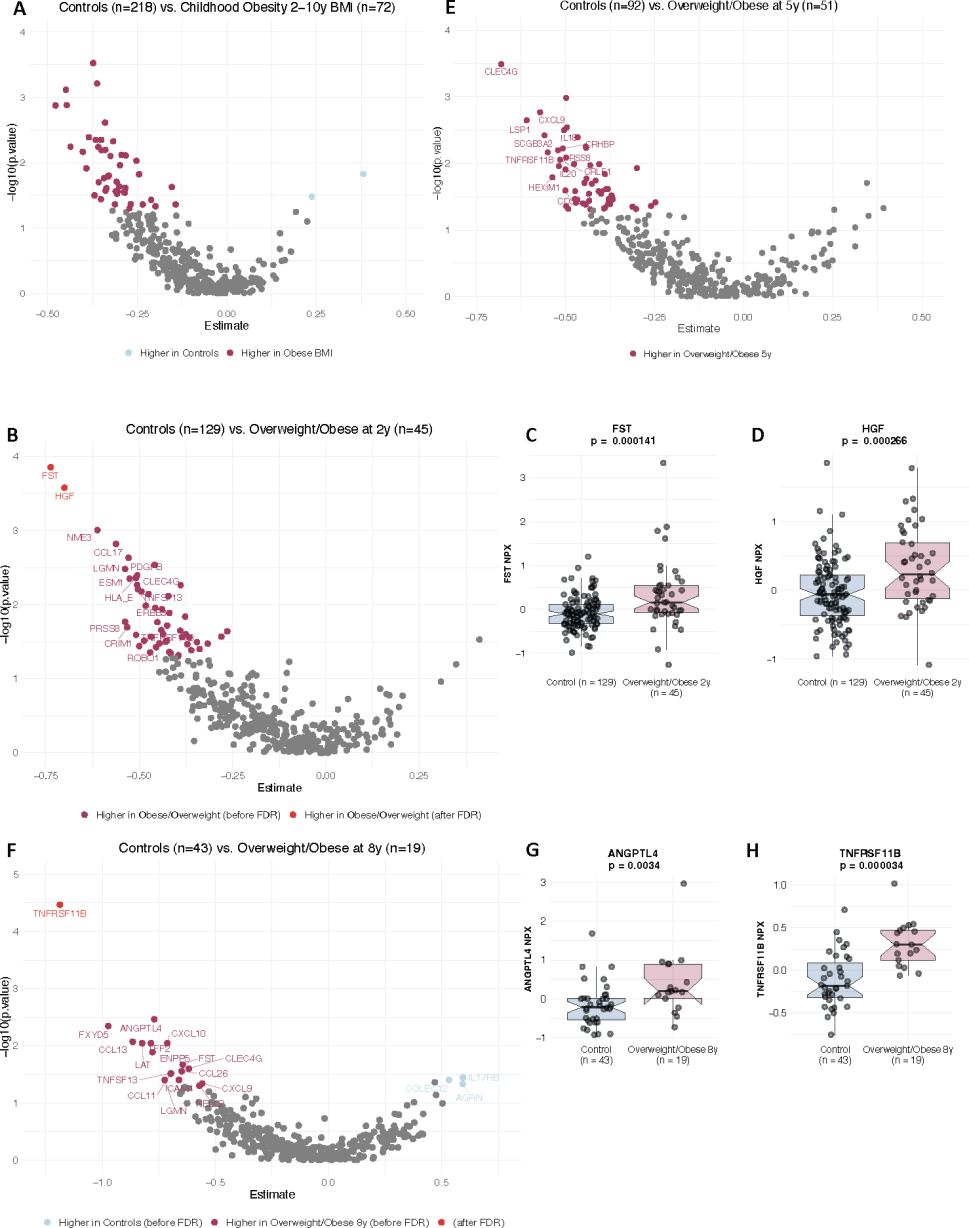
Proteomic differences in cord blood based on childhood BMI classification. Wilcoxon test statistics comparing NPX values of proteins in cord blood, based on BMI classifications across time. Controls are defined as normal-weight by IOTF standards, based on a parent-completed questionnaire, and with no future autoimmune or neurodevelopmental diagnosis from the Swedish National Patient Register. Comparisons include the following: 2–10 years global comparison (218 controls vs. 72 a BMI corresponding to obesity at least once across the timepoints), BMI at 2 years (129 controls vs. 45 overweight/obese), BMI at 5 years (92 controls vs. 51 overweight/obese), and BMI at 8 years (43 controls vs. 19 overweight/obese). (**A**) Children with BMIs indicative of obesity per IOTF standards at any point between 2 and 3, 5, 8, or 10 years of age were compared to controls using Wilcoxon tests with FDR correction. (**B–H**) Children classified as overweight or obese per the IOTF definition were compared to controls: (**B–D**) at age 2 years, (**E**) at age 5 years, (**F–H**) at age 8 years. No proteomic findings in panels** A** or **E** reached significance after FDR correction. See Table S1 for statistics.

Proteomic differences were analyzed across BMI classifications at 3, 5, 8, and 10 years (Table S1) by comparing 72 obese children with 218 controls. Across all ages, 48 proteins were significant, but none after FDR correction (Fig. 4A). In the 2- to 3-year group (45 overweight/obese vs. 129 controls), 52 proteins were significant, 2 of which (follistatin [FST] and hepatocyte growth factor [HGF]) remained significant after FDR (Fig. 4B through D). At 5 years (51 overweight/obese vs. 92 controls), 59 proteins were identified, but none survived FDR (Fig. 4E). FST and HGF were still elevated, albeit with a weaker association. At 8 years (19 overweight/obese vs. 43 controls), 21 proteins were significant, with TNFRSF11B remaining after FDR correction and higher in the overweight/obese group (*P* = 0.000034; Fig. 4F through H). Elevated ANGPTL4 was also notable (*P* = 0.0034). At 10 years (21 overweight/obese vs. 54 controls), 39 proteins were identified, but none were significant after FDR.

**Fig 5:**
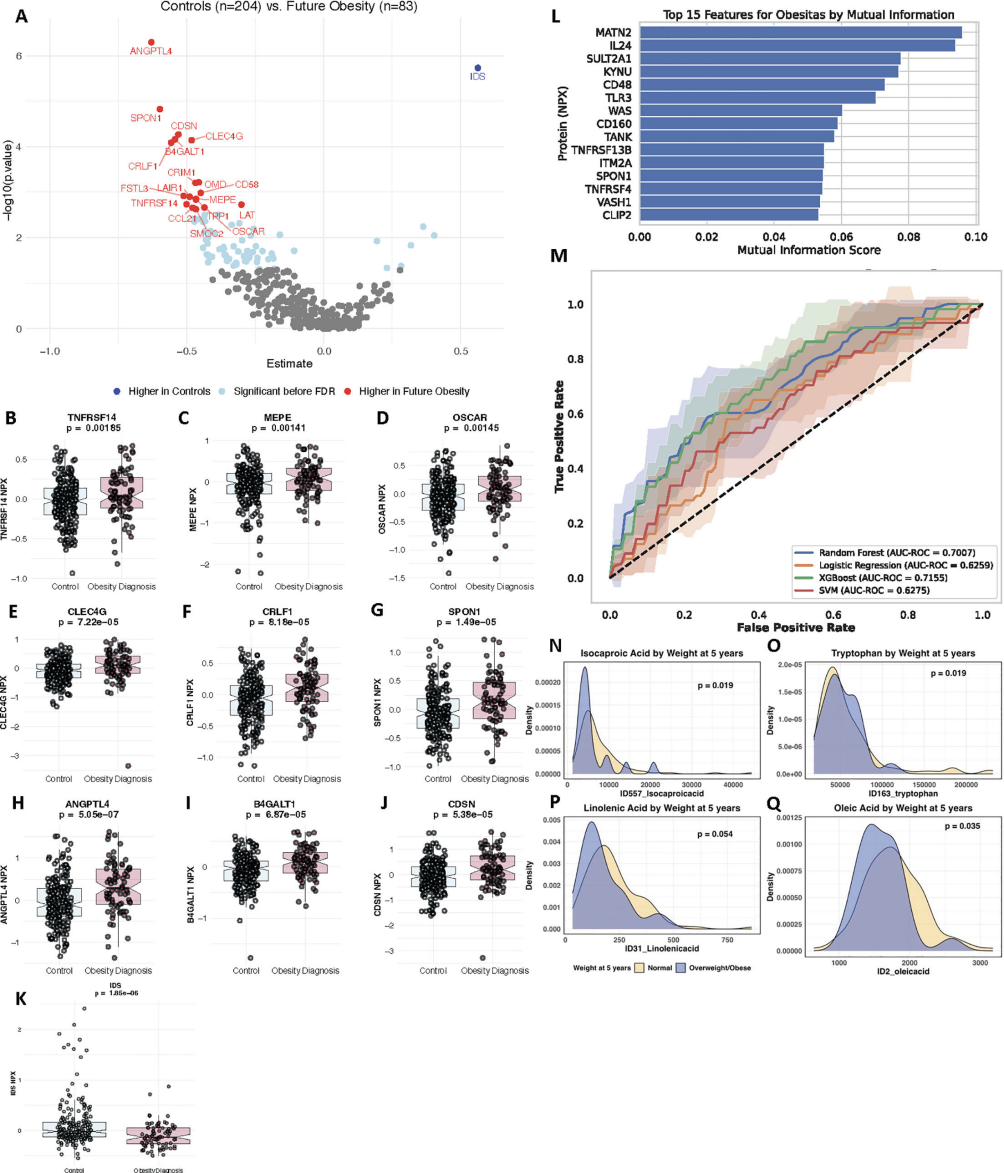
Birth proteome and metabolome associations with future clinically diagnosed obesity. (**A**) Wilcoxon test results for cord blood protein levels in relation to future ICD-10 diagnosis of clinical obesity (identified in the Swedish National Patient Register), corrected by FDR correction. (**B–K**) NPX levels for top hits, stratified by future obesity diagnosis case status. See Table S2 for statistics. (**L**) Top 15 proteins contributing to case–control discrimination of obesity (obesitas), based on mutual information (MI) scores. (**M**) Predictive performance of ML and logistic regression models predicting future obesity diagnosis using the top 15 proteins from panel K. (**N**) Improved AUC-ROC (means and standard deviation) after incorporating four additional proteins—ANGPTL4, FST, HGF, and TNFRSF11B—identified in earlier obesity comparisons. (**O–P**) Concentrations of cord serum metabolites differed between normal-weight and overweight infants, with density curves representing the concentration distributions.

Among newborns who later received a clinical diagnosis of obesity (up to 26 years later), 84 proteins were differentially expressed, with 19 remaining significant after FDR correction (Fig. 5A and Table S2). All but one of these 19 were more elevated in the obesity group (Fig. 5B through K), while IDS was the lone protein higher in controls at birth. Although mother’s weight was associated with ANGPTL4 expression (*P* = 1.547e−05), significance remained after controlling for this factor (*P* = 3.45e−06), with no interaction effect found in ANCOVA (*P* = 0.221).

Using mutual information (MI) scoring, 15 proteins were associated with clinical obesity prediction (Fig. 5L). An XGBoost model for obesity measurement at age 19 years achieved an AUC-ROC of 0.67 (Fig. S2A) and the histogram-based gradient boosting classification tree an AUC of 0.66 for a future diagnosis of obesity (Fig. S2B), with the highest performing model being the XGBoost approach with an AUC of 0.71 (Fig. S2C). Including four additional proteins (ANGPTL4, FST, HGF, and TNFRSF11B) identified in the previous analyses improved the AUC-ROC to 0.71 (Fig. 5M).

### Metabolomic differences at birth associated with obesity at age 5 years

Cord serum metabolite concentrations were compared between the normal-weight group (*n* = 132) and a group of overweight or obese children at 5 years of age (*n* = 22). Isocaproic acid (*P* = 0.19; Fig. 5N), tryptophan (*P* = 0.019; Fig. 5O), and oleic acid (*P* = 0.035, Fig. 5Q) were significantly lower in infants who were later overweight/obese.

### Predicting metabolites and exogenous compounds in cord blood from prenatal/perinatal features

ML models were developed to predict concentrations of fatty acids, bile acids, amino acids, and environmental toxins (Fig. S3A and B) from prenatal and perinatal factors, assessing which early-life factors contain predictive information about the cord blood composition. Table S3 presents the top features predicted by cord serum, selected for their predictive performance, achieving a balance between minor and major class accuracy. SHapley Additive exPlanation values were used to interpret which features contribute most to the model’s predictions, not to establish causal relationships. Thus, the identified associations represent predictive patterns rather than causal effects. Among the most predictable features with the highest accuracies included delivery mode, week of delivery, and parity, as well as mother’s diet during pregnancy, such as coffee consumption, dairy intake, coffee bread consumption, and the type of fat used on sandwiches, as well as maternal smoking during pregnancy three months prior to conception. Children born to mothers who smoked had higher 2-hydroxyacetophenonesulfate [*n* = 42; Kruskal–Wallis χ² (1) =45.7 *P* = 1.394e−11], with an average peak intensity of 9.35 (SD = 8.8), compared to 2.00 (SD = 3.4) in controls (Fig. S4A). The prediction accuracy for smoking exposure (AUC = 0.80 ± 007; Fig. S4B) was driven primarily by 2-hydroxyacetophenonesulfate, a derivative of acetaminophen (paracetamol). This metabolite was among the most accurately predicted by prenatal factors (*R*^2^ = 0.30; Fig. S4C), including coffee intake during pregnancy (Fig. S4D and E). Delivery mode associated greatly with the cord blood metabolome (Fig. S4F and G), demonstrating the strongest AUC (0.96 ± 0.02, Fig. S4F).

### Significant microbiome associations with future clinical obesity diagnosis

**Fig 6:**
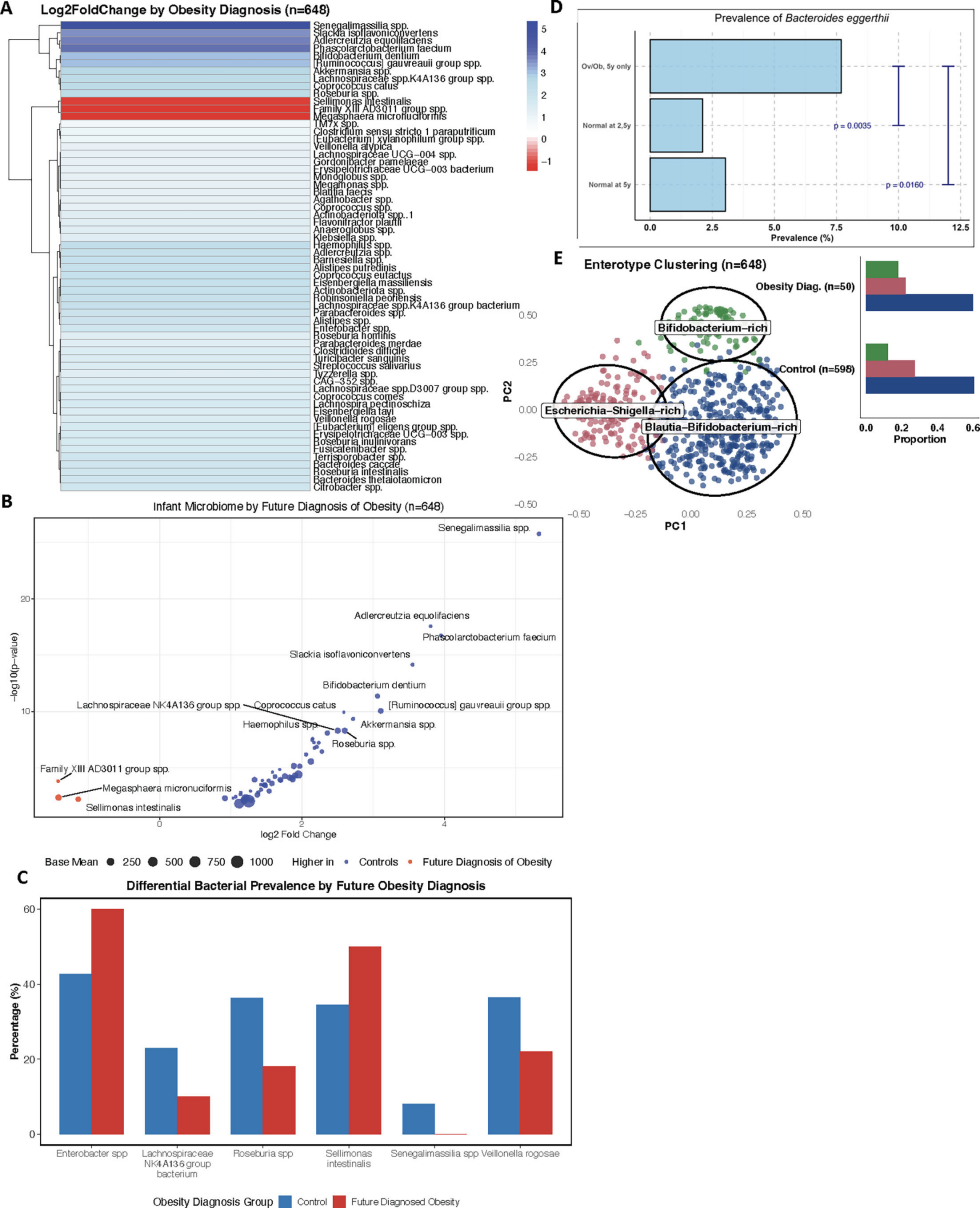
Differentially abundant or prevalent bacterial species by future diagnosis of obesity. Differentially abundant or prevalent bacterial species in future clinical diagnosis of obesity. (**A and B**) Significant differences in bacterial species abundance, after FDR correction. Controls were defined as those without obesity at the 2–3 years and 5 years timepoints, based on their BMI classifications according to the IOTF standards, as well as no future autoimmune or neurodevelopmental diagnosis. A total of 598 controls were compared to 50 future clinical obesity cases. The 16s rRNA sequencing data were assessed for differentially abundant species between obesity and control groups using DESeq2, with a binomial distribution employed for modeling the counts and using a local fit type to adjust for dispersion differences within the data set. This is important for the heterogeneous variance seen in these data sets. Multiple testing was adjusted for using the FDR method. (**C**) Significant differences in the prevalence of bacterial species. Chi-square tests assessed differences in bacterial prevalence between control and obesity groups, with Fisher’s exact tests used for taxa with low counts. For each significant taxon, prevalence and percentage presence/absence were calculated for each group. (**D**) Differences in the prevalence of *Bacteroides eggerthii*. (**E**) Enterotypes representative of obesity diagnosis and control groups, and the proportion of groups belonging to each.

*Sellimonas intestinalis*, *Family XIII AD3011 group* spp., and *Megasphaera micronuciformis* were elevated in children who were later diagnosed with obesity, whereas *Senegalimassilia* spp., *Slackia isoflavoniconvertens*, *Adlercreutzia equolifaciens*, *Phascolarctobacterium faecium*, *Bifidobacterium dentium*, *Ruminococcus gauvreauii group* spp., and *Akkermansia* spp. were markedly depleted (Fig. 6A and B). *Lachnospiraceae NK4A136 group bacterium*, *Veillonella rogosae*, *Roseburia* spp., and *Senegalimassilia* spp. showed reduced prevalence in children later diagnosed with obesity. *Senegalimassilia* spp. was entirely absent in this group (Fig. 6C). Conversely, *Enterobacter* spp. and *Sellimonas intestinalis* were more prevalent (Fig. 6C).

Among the species more abundant in matched controls (those without weight issues at ages 2–3 or 5 years) were *Phascolarctobacterium faecium, Akkermansia* spp., *Coprococcus comes, Coprococcus eutactus, Slackia isoflavoniconvertens, Bacteroides thetaiotaomicron, Parabacteroides merdae, Senegalimassilia* spp., and *Bifidobacterium dentium. Senegalimassilia* spp. was detected in only one case at both age 3 and 5 (1.75% prevalence compared to 9.97% in matched controls). Comparisons of the full data sets and the propensity score-matched sample sets are provided in Table 2 to demonstrate covariate balance.

**TABLE 2:** Baseline characteristics before and after propensity score matching for differential abundance testing^*a*^

Parameter	Comparisons (SD)							
	Obesity 2 yrs (all)		Obesity 2 yrs (propensity)		Obesity 5 yrs (all)		Obesity 5 yrs (propensity)	
	0	1	0	1	0	1	0	1
*n*	648	13	39	13	501	22	66	22
BMI pre-pregnancy (mean)	24.2 (3.9)	26.2 (4.3)	25.9 (4.3)	26.2 (4.3)	24.3 (4.0)**	26.3 (4.0)**	25.9 (4.8)	26.3 (4.0)
Region (%)								
East	146 (23)	2 (15)	9 (23)	2 (15)	106 (21)	2 (9.1)	5 (7.6)	2 (9.1)
North	180 (28)	5 (39)	19 (49)	5 (39)	140 (28)	9 (41)	25 (38)	9 (41)
South	158 (24)	2 (15)	2 (5.1)	2 (15)	130 (26)	6 (27.3)	23 (35)	6 (27)
West	164 (25)	4 (31)	9 (23)	4 (31)	125 (25)	5 (23)	13 (20)	5 (23)
Gastroenteritis in 1st year (yes, %)								
1 = yes	185 (29)	6 (46)	16 (41)	6 (46)	142 (28)	8 (36)	23 (35)	8 (36)
2 = no	447 (69)	7 (54)	22 (56)	7 (54)	345 (69)	14 (64)	42 (64)	14 (64)
3 = unk	16 (2.5)	0 (0.0)	1 (2.6)	0 (0.0)	14 (2.8)	0 (0.0)	1 (1.5)	0 (0.0)
Total breastfeeding, months (%)								
1	24 (3.7)	1 (7.7)	1 (2.6)	1 (7.7)	17 (3.4)	0 (0.0)	5 (7.6)	0 (0.0)
2	25 (3.9)	2 (15)	3 (7.7)	2 (15)	18 (3.6)	1 (4.5)	2 (3.0)	1 (4.5)
3	30 (4.6)	0 (0.0)	2 (5.1)	0 (0.0)	21 (4.2)	1 (4.5)	4 (6.1)	1 (4.5)
4	20 (3.1)	0 (0.0)	2 (5.1)	0 (0.0)	19 (3.8)	0 (0.0)	3 (4.5)	0 (0.0)
5	25 (3.9)	0 (0.0)	2 (5.1)	0 (0.0)	21 (4.2)	2 (9.1)	4 (6.1)	2 (9.1)
6	59 (9.1)	0 (0.0)	3 (7.7)	0 (0.0)	42 (8.4)	4 (18.2)	4 (6.1)	4 (18)
7	70 (11)	2 (15)	2 (5.1)	2 (15)	51 (10)	3 (14)	10 (15)	3 (14)
8	104 (16)	4 (31)	6 (15)	4 (31)	79 (16)	6 (27)	6 (9.1)	6 (27)
9	291 (45)	4 (31)	18 (46)	4 (31)	233 (46)	5 (23)	28 (42)	5 (23)
Infection with antibiotics in first year (%)								
1 = none	435 (67)	2 (15)	12 (31)	2 (15)	342 (68)	14 (64)**	52 (79)**	14 (64)
2 = 1–2×	175 (27)	9 (69)	13 (33)	9 (69)	130 (26)	8 (36)	12 (18)	8 (36)
3 = 3–5×	36 (5.6)	2 (15)	12 (31)	2 (15)	29 (5.8)	0 (0.0)	2 (3.0)	0 (0.0)
4 = more	2 (0.3)	0 (0.0)	2 (5.1)	0 (0.0)				
Gestational age, weeks (%)								
33	2 (0.3)	0 (0.0)			2 (0.4)	0 (0.0)	1 (1.5)	0 (0.0)
34	1 (0.2)	0 (0.0)			2 (0.4)	0 (0.0)		
35	6 (0.9)	0 (0.0)			4 (0.8)	0 (0.0)	1 (1.5)	0 (0.0)
36	14 (2.2)	0 (0.0)	1 (2.6)	0 (0.0)	13 (2.6)	0 (0.0)	1 (1.5)	0 (0.0)
37	36 (5.6)	0 (0.0)	2 (5.1)	0 (0.0)	24 (4.8)	3 (13.6)	1 (1.5)	3 (14)
38	58 (9.0)	1 (7.7)	2 (5.1)	1 (7.7)	44 (8.8)	2 (9.1)	2 (3.0)	2 (9.1)
39	133 (21)	3 (23)	6 (15)	3 (23)	112 (22)	1 (4.5)	18 (27)	1 (4.5)
40	185 (29)	2 (15)	12 (31)	2 (15)	144 (29)	4 (18)	15 (23)	4 (18)
41	133 (21)	4 (31)	8 (21)	4 (31)	110 (22)	8 (36)	16 (24)	8 (36)
42	67 (10)	3 (23)	6 (15)	3 (23)	41 (8.2)	4 (18)	10 (15)	4 (18)
43	13 (2.0)	0 (0.0)	2 (5.1)	0 (0.0)	5 (1.0)	0 (0.0)	1 (1.5)	0 (0.0)
Delivery mode (C-section, %)	74 (11)	3 (23)	10 (26)	3 (23)	59 (12)	4 (18)	11 (17)	4 (18)
Sex (female, %)	316 (49)	7 (54)	26 (67)	7 (54)	231 (46)*	16 (73)*	49 (74)	
Vulnerability index 4 (%)								
0 = low	321 (50)	6 (46)	16 (41)	6 (46)	243 (49)	8 (36)	19 (29)	8 (36)
1	241 (37)	5 (39)	16 (41)	5 (39)	198 (40)	10 (46)	35 (53)	10 (46)
2	69 (11)	2 (15)	6 (15)	2 (15)	51 (10)	2 (9.1)	11 (17)	2 (9.1)
3 = high	17 (2.6)	0 (0.0)	1 (2.6)	0 (0.0)	9 (1.8)	2 (9.1)	1 (1.5)	2 (9.1)

^*a*^ Comparisons between groups were performed using the CreateTableOne package in R. By default, continuous variables were compared using a *t*-test (mean ± SD reported for BMI pre-pregnancy), and categorical variables were compared using a chi-square test, with Fisher’s exact test used for variables with expected cell counts <5. Only significant *P*-values below 0.05 are shown. ***P = 0.001, **P = 0.021. *P = 0.026, unk, unknown.

In contrast, *Bacteroides eggerthii* was more abundant in infants who later had weight issues. The bacterium was seen in only 2.1% of infants with normal weight at both 3 and 5 years, but in 7.7% of infants later classified as obese or overweight at 5 years [χ² (1) =8.5, *P* = 0.0035; Fig. 6D]. Similarly, *ASV-103 Klebsiella* sp. was more abundant in infants who later became obese at ages 3 or 5 years. This strain was detected in 14.2% and 11.0% of infants with normal weight or overweight at 3 years, respectively, but in 35.5% of cases. At age 5, it was detected in 12.7% of infants with normal weight, compared to 19.7% overweight and 26.2% obese at this age. Although no enterotype-level differences were found based on future diagnosis (Fig. 6E), species-level distinctions suggest more precise differences in the microbiome significant for predicting future weight issues.

To address potential confounding factors influencing microbiome composition, a strict iteration of propensity score matching was applied in a 3:1 ratio using nearest neighbor. This matching included gestational diabetes, the mother’s pre-pregnancy BMI, geographic location, gastroenteritis in the first year, total months of breastfeeding, antibiotic exposure during the first year, mode of delivery, biological sex, and psychosocial vulnerability. The psychosocial vulnerability score was calculated from a set of variables including living conditions, parental employment and education, serious stressful life events, disposable income, alcohol, and smoking (21). This adjustment revealed 26 significant taxa when comparing 39 controls against 13 cases at age 2–3, and 30 taxa for cases at age five (22 cases vs. 66 controls), defined by an FDR-adjusted *P* < 0.05 and a normalized base mean of abundance ≥10.

### Prediction of future clinical obesity diagnosis using multi-omics in the first year of life

Among 1,877 ABIS individuals (including 114 cases with a future clinical obesity diagnosis), microbiomic and/or proteomic data from early life were available. These data were used to predict future clinically diagnosed obesity using eXtreme Gradient Boosting. Machine learning models incorporated L1 regularization, class-imbalance weighting, and stratified fivefold cross-validation. Metadata were integrated to account for possible interactions between biological and non-biological features. Clinical obesity diagnosis was defined exclusively using registry-confirmed diagnoses from the Swedish National Patient Register.

To prevent information leakage, all preprocessing and feature selection steps were performed independently within each cross-validation fold. Specifically, RFE was applied using only the training data in each fold to select the top 40 predictors, enhancing model interpretability, after which models were trained on these features and evaluated on the corresponding held-out test set. Median imputation was likewise performed separately within testing and training partitions. In total, 683 candidate features were evaluated per fold, including 363 proteomic markers and 230 bacterial taxa, along with questionnaire data from birth and 1-year surveys, and calculated BMI scores up to age 8 (for the child and parents).

**Fig 7:**
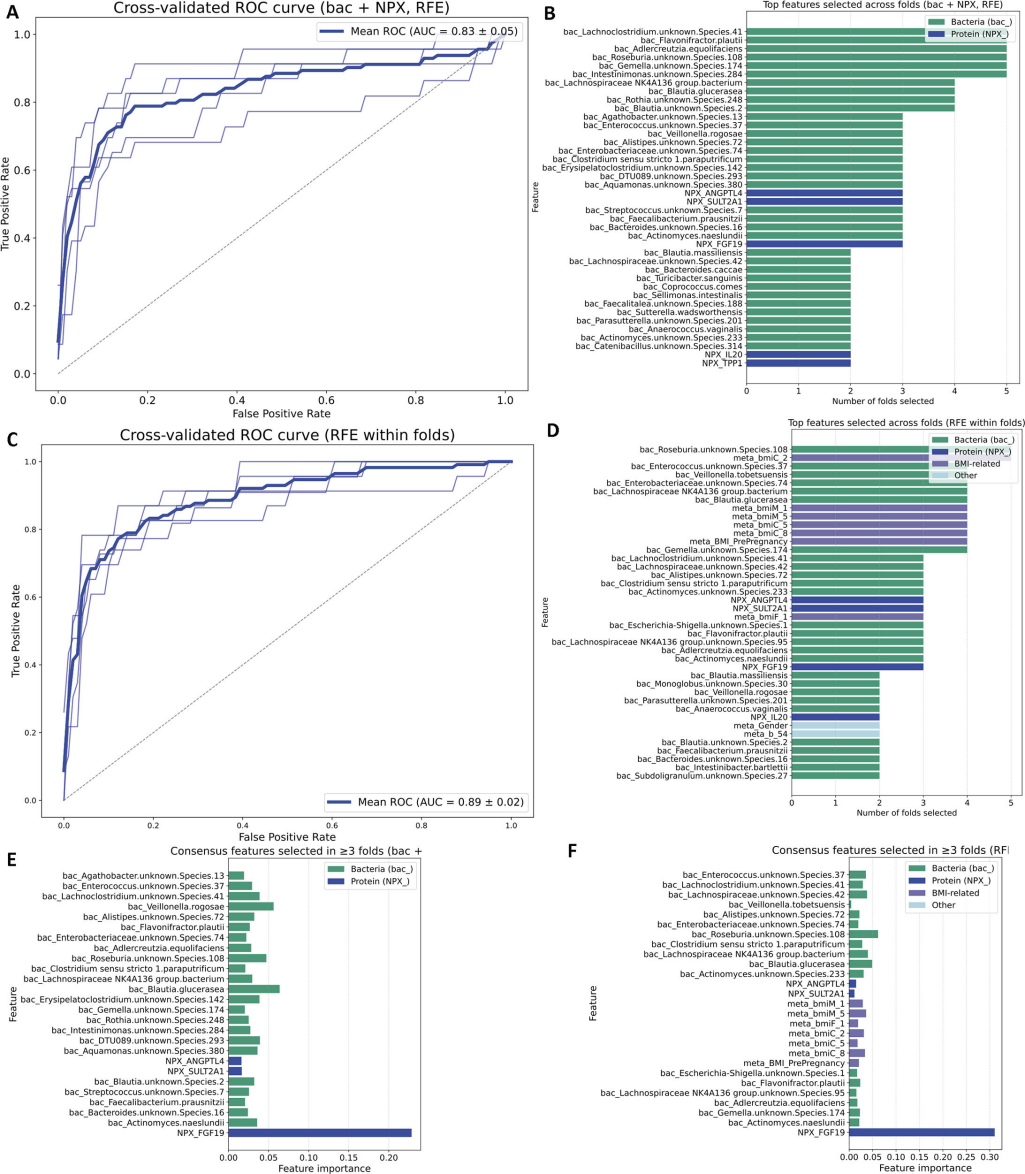
Integrated machine learning models predicting future clinical diagnosis of obesity. Prediction of clinical obesity diagnosis in 1,877 individuals, based on the XGBoost Classifier with an 80/20 train/test split and RFE integrated within fivefold stratified cross-validation. The primary outcome was clinical diagnosis in the Swedish NPR (22). The integrated predictive models relied exclusively on registry-confirmed clinical obesity diagnoses as the primary outcome, thereby avoiding dependence on self-reported anthropometric measurements. Panels **A** and **B** show predictions based on proteins at birth and taxa present in one-year stool samples, while panels **C** and **D** additionally incorporate metadata from the birth and 1-year questionnaires. For each fold, the training set was used to select the top 40 features via RFE (from a total of 683 features, including 230 bacterial and 363 proteomic). The model was subsequently trained on these features and evaluated on the held-out test set. L1 regularization (alpha = 1) and class weighting (scale_pos_weight) were applied to handle potential overfitting and class imbalance. Missing values were handled using median imputation fit on the training data within each fold and then applied to the corresponding test set, preventing information leakage across folds. Performance metrics—including ROC AUC, F1 score, precision, and recall—were averaged across the folds. The top features selected across folds were summarized by counting the number of times each feature was chosen. The selected features indicate patterns associated with obesity risk rather than independent causal effects. AUC average and standard deviation across folds are shown in panels **A** and** C**, with feature importances for the top features in panels** B** and **D**. Feature naming conventions are as follows: bacterial features begin with “bac_,” while proteomic birth features begin with “NPX_.” Environmental and anthropometric variables include meta_b_54, corresponding to dietary vegetable intake frequency at 1 year of age; maternal BMI measures (meta_BMI_PrePregnancy, meta_bmiM_1, meta_bmiM_5); paternal BMI at 1 year (meta_bmiF_1); and child BMI at ages 2–3, 5, and 8 years (meta_bmiC_2, meta_bmiC_5, and meta_bmiC_8). (**E**) Feature importance of consensus predictors selected in at least three cross-validation folds. Features retained in every fold (consensus features) are shown, with colors indicating feature type: proteins (NPX_, blue), bacteria (bac_, green), and other metadata features (gray). Feature importance values represent the contribution of each feature to a final XGBoost model predicting future obesity diagnosis.

Models integrating both microbiomic and proteomic data achieved a mean ROC AUC of 0.83 ± 0.05 (Fig. 7A) with a mean F1 score of 0.53 ± 0.06. At the selected classification threshold, mean precision (0.51 ± 0.06) indicated that approximately half of the individuals predicted to develop obesity were true positives, while mean recall (0.56 ± 0.10) demonstrated that the model correctly identified over half of all true future obesity cases. These results indicate robust discriminative performance while maintaining a balance between sensitivity and specificity. The most predictive feature was FGF19 in cord blood, followed by ANGPTL4 and SULT2A1 as proteomic markers at birth (Fig. 7B and E) and consensus bacterial markers at age 1 (identified in ≥3 folds), including *Blautia glucerasa*, *Veillonella rogosae*, *Roseburia* spp., *Erysipelatoclostridium* spp., *Actinomyces naeslundii*, and *Lachnoclostridium* spp. (Fig. 7B and E).

In models additionally incorporating early-life metadata and early-life BMI metrics, discriminative performance improved slightly further, achieving a mean ROC AUC of 0.89 ± 0.02, indicating strong overall discriminative performance (Fig. 7C). Feature stability across folds was summarized by counting the number of times the feature was chosen (Fig. 7D). The most frequently selected proteomic predictors included ANGPTL4, SULT2A1, FGF19, and IL20 (Fig. 7D), while predictive bacterial included *Roseburia* spp., *Enterococcus* spp., *Veillonella tobetsuensis*, *Enterobacteriaeae* spp., *Lachnospiraceae NK4A136* group, *Blautia glucerasea*, and *Gemella* spp. (Fig. 7D). Parental BMI (pre-pregnancy and during early childhood) and child BMI at ages 2–3, 5, and 8 years also emerged as consistently informative features (Fig. 7D). Among consensus predictors (identified in at least three of the five folds), FGF19 exhibited the highest feature importance (score > 0.3), followed by *Roseburia* spp., *Blautia glucerasea*, Lachnospiraceae spp., Enterococcus spp., Lachnospiraceae NK4A136 group, the BMI metrics (from mother, father, and child), and ANGPTL4 (Fig. 7E).

Accuracy metrics for both the Bacterial +Proteomic and the Bacterial +Proteomic + Metadata models are provided in Table S4. Together, these findings indicate that, even across a 26-year predictive gap, cord blood protein profiles, early-life bacterial profiles, and parental anthropometrics provide stable and biologically informative signals associated with long-term obesity risk.

## DISCUSSION

Studying early-life biological markers associated with metabolic disorders and obesity can identify targets useful in future studies for the prediction and prevention of these disorders. While other prospective studies (18, 28–32) have explored these links, this is the first to examine microbial differences at age 1 year, alongside metabolomic and proteomic differences at birth, using integrated prediction models powered by interpretable algorithmic machine learning methods. This focus allows identification of critical markers of early-life programming, long before clinical outcomes manifest.

The microbiome is influenced by many well-studied factors, some of which were controlled for here, including breastfeeding practices, antibiotic use (19, 33–35), gastroenteritis, maternal gestational diabetes, pre-pregnancy weight, and psychosocial factors. Parental obesity increases the risk of a child becoming obese as an adult (5). Infants born to obese mothers have reduced *Proteobacteria* and increased *Coriobacteriaceae*, *Erysipelotrichaceae*, *Lachnospiraceae*, and *Ruminococcaceae* (36). Children born to an overweight mother by cesarian section are at the greatest risk of obesity between ages 1 and 3 years, with increased abundance of *Lachnospiraceae* possibly associated with changes in weight (36).

*Akkermansia muciniphila* is associated with immune system regulation (37), mucin health, anti-inflammatory processes (38–40), and metabolic status (8, 16, 41, 42). Here, *Akkermansia* spp. were less abundant in infants with future obesity. Conversely, *B. eggerthii*, known to promote colitis (43), and possessing a polysaccharide-utilizing locus (44), was higher. Asaccharolytic bacteria, that is, unable to metabolize sugars, *Senegalimassilia* and *Phascolarctobacterium,* were more abundant in controls, while equol-producing bacteria *Slackia* and *Adlercreutzia* were higher in infants with future obesity, which fits the previous observation that equol has been associated with lower BMI, fat mass percentage, and serum triglycerides in a community-based prospective study of adults (45). Equol and equol-producing microbes have also been favorably associated with other obesity markers like liver fat (46). *Klebsiella* ASV-103, more prevalent in infants with future weight problems, is the same strain that we found more abundant in infants with future neurodevelopmental disorders (19), often co-occurring with obesity (47).

Among the most significantly elevated proteins at birth in those with future obesity were FST, HGF, and ANGPTL4. Circulating FST has been shown to be elevated in patients with type 2 diabetes, potentially due to adipose insulin resistance and metabolic dysregulation (48). HGF, which plays a key role in insulin resistance through the HGF/c-Met signaling pathway (49), is elevated in conditions associated with insulin resistance (50) and obesity (51). ANGPTL4, a protein studied with respect to metabolic and cardiovascular diseases (52), is induced by fasting and lipid signals. ANGPTL4 is secreted by many cell types, including macrophages, and plays critical roles in energy homeostasis and lipid metabolism (53). Knockout models exhibit enhanced clearance of triacylglycerol-rich lipoproteins, reduced inflammation, and improved glucose metabolism (54). The association of ANGPTL4 with future obesity outcomes, independently of maternal weight status, highlights the potential of early-life disturbances in metabolism as independent risk factors, separate from maternal influences.

Where stronger associations with BMI at ages 2–3 years occur, such as the FST and HGF proteins, this may be owing to a clinical diagnosis closer to sampling. We hypothesize that attenuation of associations with BMI at later time points (e.g., only one marker reaching FDR significance at age 8) is due to smaller sample sizes and increased variability in BMI reporting. Despite these limitations, our findings suggest that clinically diagnosed obesity provides a more reliable, biologically grounded phenotype with stronger proteomic correlates.

We found that the metabolome strongly associates with delivery mode, preterm birth, and whether the child went to a “newborn ward,” which generally refers in Sweden to a neonatal intensive care unit (NICU), providing specialized care for newborns. Branched-chain amino acids (BCAAs), medium- and long-chain acylcarnitines, nonesterified fatty acids, and triglycerides have been positively associated with birth weight (55). These are negatively associated with C-peptide, a byproduct of insulin secretion that impacts maternal and fetal health (56, 57). Cholestenone and BCAAs have been previously identified as predictors of rapid growth and overweight/obesity in newborns (58), with lysophosphatidylcholines and glycerophospholipid fatty acids (GPL-FA) related to birth weight (59). Prenatal factors like maternal education, pre-pregnancy BMI, weight gain during pregnancy, tobacco smoke exposure, maternal diet, delivery mode, and birth order influence the composition of cord blood metabolites, consistent with past studies (60–62). In a smaller study, aligned with our findings of the metabolome’s strong association with parity, birth order correlated with histidine metabolism and the tricarboxylic acid cycle; sex was associated with BCAA metabolism and vitamin B6 metabolism; whereas mode of delivery was associated with glyoxylate and dicarboxylic acid metabolism (61). Higher intake of margarine or soy oil was associated with E- and Z-octadecanoic acid, hydroxyphenylacetic acid, and uric acid, which are metabolites predictive of allergies (63). One of the most predictable features was whether the mother smoked during pregnancy, which has been linked to alterations in cord blood metabolites from lipid metabolism, such as phosphatidylcholines and sphingomyelins (64). Offspring DNA methylation significantly associates with nicotine and its downstream metabolites in maternal and cord sera (65), with MYO1G, AHRR, and GFI1 genes implicated in cardiovascular complications, as are advanced oxidation protein products in the cord blood (62). We previously reported on the adverse consequences of maternal smoking in our study of neurodevelopment (19). These associations offer explanations for the metabolic profiles observed in the cord blood and possible areas for intervention in pregnancy.

Limited studies have explored the predictive value of early, integrated omics for future obesity risk (36). Here, bacterial and proteomic markers measured within the first year of life provided an unprecedented opportunity to model long-term prediction accuracy for clinical obesity diagnoses many years later using a base 40-marker microbiome and proteomic panel. Among the top proteomic predictors were fibroblast growth factor 19 (FGF19), angiopoietin-like 4 (ANGPTL4), sulfotransferase family 2A member 1 (SULT2A1), and interleukin 20 (IL20), proteins that converge on bile acid metabolism, lipid handling, and immune regulation.

FGF19 and SULT2A1 are centrally involved in bile acid metabolism and signaling. FGF19, an enterohepatic hormone primarily produced in the ileum, regulates carbohydrates, glucose homeostasis, and bile acid synthesis (66), and SULT2A1 is significant for the elimination and detoxification of bile acids as well as the sulfation of steroid hormones, altering their bioavailability and activity (67). FGF19 is induced by bile acid signaling. Because bile acid availability and composition are strongly shaped by the gut microbiome (52), altered levels of these proteins at birth may reflect early-life bile acid signatures that contribute to later metabolic risks. ANGPTL4 further supports this axis, as it inhibits intestinal lipase activity (68), suggesting mechanisms of fat accumulation and insulin resistance. Importantly, ANGPTL4 facilitation of bile acid absorption is gut microbiota-dependent (69), which underscores the interaction between ANGPTL4 and the key bacteria identified in our integrated model. ANGPTL4 has been considered for cardiometabolic therapies, studied most recently for its roles in glucose intolerance (70). Bile acid composition and amounts are largely shaped by the gut microbiome (71, 72). Here also, oleic acid levels were depleted at birth in children who were later overweight or obese. Bile acids are inversely associated with abdominal fat, possibly due to downregulation of inflammatory responses (73). The consensus bacterial markers, including *Roseburia* spp., *Enterococcus* spp., *Veillonella tobetsuensis*, *Lachnospiraceae NK4A136 group*, *Blautia glucerasea*, and *Gemella* spp. are notable for their significance to bile acid pool composition, short-chain fatty acid (SCFA) production, and carbohydrate utilization. In addition, B4GALT1, which was significantly higher in future cases, plays key roles in synthesizing glycoproteins and lactose metabolism (74), further supporting altered carbohydrate-related pathways in early life. Meanwhile, IL20, a critical pro-inflammatory cytokine involved in immune modulation (75), mediating immunity and epithelial tissue health, may reflect early immune tone relevant to metabolic health. It is involved in the dysregulation of macrophages and modulates adipogenesis in *in vitro* models, demonstrating a possible target (76). Consistent with this immune-metabolic interface, the consensus bacterial marker *Enterobacteriaceae* spp. is associated with lipopolysaccharide (LPS) production and low-grade inflammation (77).

Together, these results provide evidence that cord blood proteomic signatures encode biologically meaningful information about future obesity risk. Although precision and recall illustrate the challenges inherent in long-term prediction, the models’ AUC and F1 scores present strong signals from birth, demonstrating the reliability of these markers. The association, for instance, of ANGPTL4 being independent of maternal weight, supports the idea that markers reflect intrinsic biological risk rather than simply mirroring maternal adiposity downstream. Consistent with prior literature (5), parental BMI in the child’s early life remained an important predictor; however, the integrated model highlights the additional and complementary contribution of early-life biological signatures to long-term obesity risk.

Our findings present the earliest timepoints of microbial dysbiosis to study risk for childhood obesity. The discovery of proteomic and microbiome markers at birth and in infancy, long before the onset of obesity, also underscores the importance of early metabolic programming. While our models show strong discrimination (AUC ~ 0.89), we note that predicted probabilities should not be interpreted as calibrated clinical risk estimates without external validation. These probabilities are not yet calibrated for clinical use. The models are optimized for ranking/classification rather than probability calibration. Future work will assess calibration using methods such as Platt scaling or isotonic regression in external cohorts before clinical deployment. This observational study is intended to generate hypotheses for further testing. Nevertheless, identification of these early-life biomarkers may suggest early interventions to prevent disease.

### Limitations

There are several limitations to this investigation. (i) The relatively small numbers of obesity cases in some key omics contrasts should be noted (e.g., 22 overweight/obese vs. 132 normal-weight in the metabolomics comparison; 50 obesity cases vs. 598 controls in microbiome analyses), which may limit statistical power and increase uncertainty in effect estimates. (ii) Obesity diagnosis through early adulthood was observed in 2.4% of subjects (*n* = 407 of 16,683), likely indicating underdiagnosis rather than representing true population prevalence. Since clinical diagnoses of obesity often capture only the most severe cases, these approaches rely on these strict criteria, which may underestimate the broader burden of obesity. However, both registry-based clinical diagnoses and BMI were included to mitigate this potential underestimation. (iii) The omics data were not uniformly available across the cohort at the time of these analyses, such that not all individuals with clinical obesity had metabolomic, proteomic, or microbiome data ready to analyze. Different combinations of omics layers were available for subjects included in this study (e.g., proteomics without metabolomics, microbiome without proteomics), which limited sample sizes for some analyses and may affect the robustness of specific contrasts. As additional data become available, more complete multi-omic coverage will provide greater clarity regarding the stability and reproducibility of these findings. (iv) Furthermore, microbiome findings will require follow-up using shotgun metagenomic approaches to enable functional characterization and identification of microbial pathways and interactions, which will be essential for contextualizing the biological significance of the observed taxonomic differences. (v) Residual confounding is possible in both questionnaire-based and omics analyses, including unmeasured dietary factors, physical activity patterns, and genetic predisposition, which were not accounted for in the present study. In addition, generalizability may be limited beyond the Swedish population, given its specific healthcare, diet, and socioeconomic context, underscoring the importance of replication in other large cohorts. (vi) Finally, the absence of an external validation cohort for the machine learning models represents an important limitation for clinical translation. While internal cross-validation was applied, external validation will be essential in future studies to confirm robustness and portability.

## Data Availability

The processed data underlying the proteomic and microbiome analyses in this study are publicly available in the Dryad repository (https://doi.org/10.5061/dryad.2v6wwq042). The Jupyter notebook used to implement the machine learning pipelines is publicly available at https://github.com/aahren/obesity-ML-ABIS. Researchers who are interested in accessing additional data or materials underlying this work should contact the senior author, Dr. Ludvigsson. For data not available through open access, bona fide researchers may need to develop a formal collaboration with the ABIS study group for specific scientific purposes.

## SUPPLEMENTAL MATERIAL

Supplemental material msystems.01424-25-s0001.docx Supplemental text, tables, and figures.

Table S1 msystems.01424-25-s0002.xlsx Differential protein expression in cord blood by future BMI-derived obesity status during childhood.

Table S2 msystems.01424-25-s0003.xlsx Differential protein expression in cord blood by future clinical diagnosis of obesity.

ASM does not own the copyrights to Supplemental Material that may be linked to, or accessed through, an article. The authors have granted ASM a non-exclusive, world-wide license to publish the Supplemental Material files. Please contact the corresponding author directly for reuse.
