# Supplementary material for: Early-life proteomic and microbiome features signal obesity risk across 26 years of follow-up
Source: mSystems. 2026 May 28;11(6):e01424-25. doi: 10.1128/msystems.01424-25 (PMC13289108; doi:10.1128/msystems.01424-25)
Supplement: Supplemental material — Supplemental text, tables, and figures. [file msystems.01424-25-s0001.docx]

**Title**

**Early-life environment and multi-omics predict childhood and adult-onset obesity over 26 years of follow-up**

**Supplementary Materials**

**Supplementary Table 1. Differential protein expression in cord blood by future BMI-derived obesity status during childhood.** Wilcoxon test statistics comparing Normalized Protein Expression (NPX) values of proteins in cord blood, based on BMI classifications across time. Median values for each group (0 = control, 1 = case) and the effect size (Cohen’s d) between the two groups are reported. Controls are defined as normal-weight by International Obesity Task Force (IOTF) standards, based on a parent-completed questionnaire, and with no future autoimmune or neurodevelopmental diagnosis from the Swedish National Patient Register. Comparisons include: 2-10 years global comparison (218 controls vs. 72 a BMI corresponding to obesity at least once across the timepoints), BMI at 2 years (129 controls vs. 45 overweight/obese), BMI at 5 years (92 controls vs. 51 overweight/obese), and BMI at 8 years (43 controls vs. 19 overweight/obese). These statistics correspond to the plots in Fig. 3.

**Supplementary Table 2. Differential protein expression in cord blood by future clinical diagnosis of obesity.** Wilcoxon test statistics comparing Normalized Protein Expression (NPX) values of proteins in cord blood, based on future clinical diagnosis of obesity with corresponding ICD-10 codes E66.0, E66.8, and E66.9. Median values for each group (0 = control, 1 = case) and the effect size (Cohen’s d) between the two groups are reported. Controls are defined as normal-weight by International Obesity Task Force (IOTF) standards, based on a parent-completed questionnaire, and with no future autoimmune or neurodevelopmental diagnosis from the Swedish National Patient Register. A total 204 controls and 84 future obesity cases were compared. These statistics correspond to the plots in Fig. 4A-K.

**Supplementary Table 3. Top prenatal and perinatal features predicted by the neonatal metabolome.** Top-performing models (Extreme Gradient Boosting or XG Boost, Random Forest, Logistic Regression, Extra Tree) used to predict prenatal and perinatal features from the neonatal metabolome. For each model, the area under the curve (AUC) statistic is provided as mean ± standard deviation, along with the F1 score, which reflects the balance between recall and precision for major and minor classes. The comparisons made within each categorical variable are also indicated.

**Supplementary Table 4. Accuracy metrics for machine learning (ML) classification models predicting future obesity diagnosis.** Performance metrics, including mean and standard deviation across 5-fold cross-validation, as described, are reported for eXtreme Gradient Boosting (XGBoost) models with recursive feature elimination (RFE).

| **Bacterial + Proteomic ML Model** | | | | | | |
| --- | --- | --- | --- | --- | --- | --- |
| fold | roc_auc | f1 | precision | recall | n_train | n_test |
| 1 | 0.888 | 0.593 | 0.516 | 0.696 | 1501 | 376 |
| 2 | 0.804 | 0.429 | 0.474 | 0.391 | 1501 | 376 |
| 3 | 0.748 | 0.558 | 0.571 | 0.545 | 1502 | 375 |
| 4 | 0.854 | 0.481 | 0.419 | 0.565 | 1502 | 375 |
| 5 | 0.877 | 0.583 | 0.56 | 0.609 | 1502 | 375 |
| mean | 0.834 | 0.529 | 0.508 | 0.561 |  |  |
| sd | 0.052 | 0.064 | 0.056 | 0.099 |  |  |
|  |  |  |  |  |  |  |
| **Bacterial + Proteomic + Metadata ML Model** | | | | | | |
| fold | roc_auc | f1 | precision | recall | n_train | n_test |
| 1 | 0.917 | 0.615 | 0.552 | 0.696 | 1501 | 376 |
| 2 | 0.869 | 0.419 | 0.450 | 0.391 | 1501 | 376 |
| 3 | 0.881 | 0.513 | 0.588 | 0.455 | 1502 | 375 |
| 4 | 0.891 | 0.480 | 0.444 | 0.522 | 1502 | 375 |
| 5 | 0.888 | 0.468 | 0.458 | 0.478 | 1502 | 375 |
| mean | 0.889 | 0.499 | 0.499 | 0.508 |  |  |
| sd | 0.016 | 0.066 | 0.060 | 0.103 |  |  |

**Supplementary Figure 1. Proportional distribution of early-life risk factors associated with clinical obesity diagnosis.** Bar plots display the proportion of individuals with and without a future diagnosis of clinical obesity (cases and controls) who were exposed to each significant early-life risk factor identified in Fig. 1.

| ** 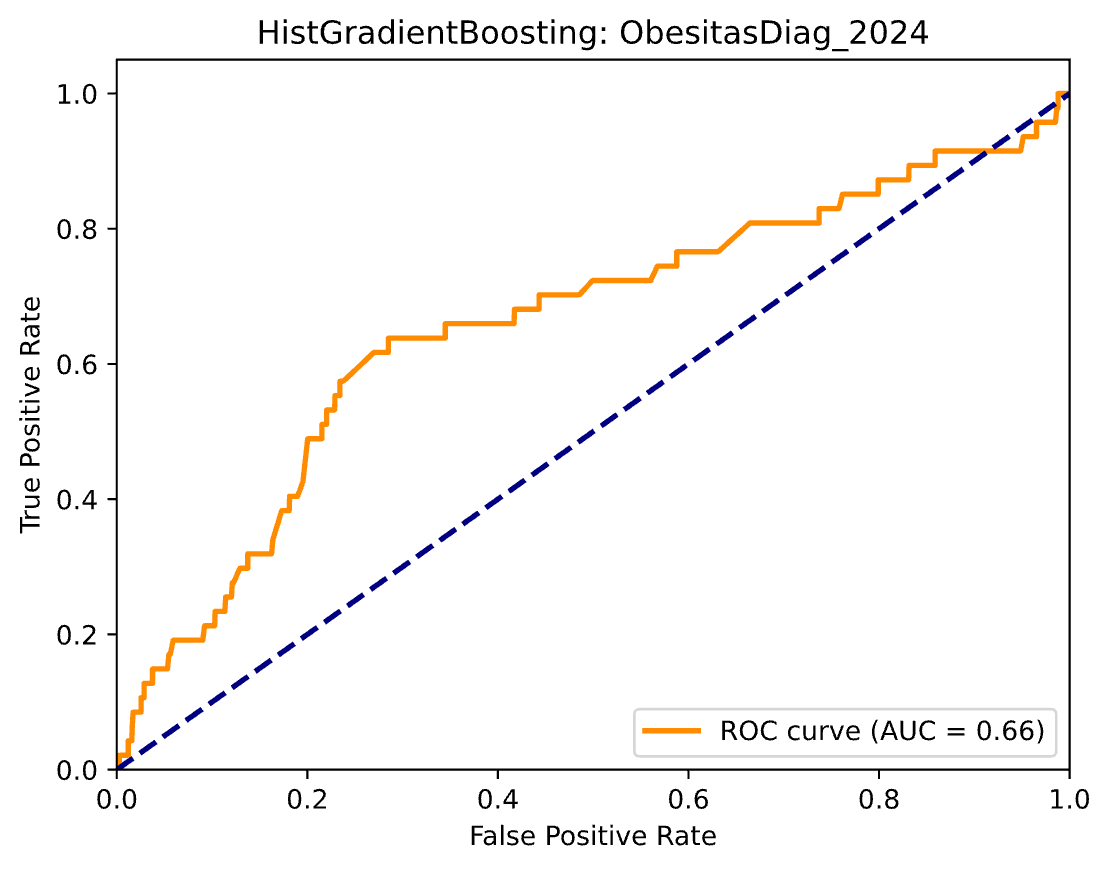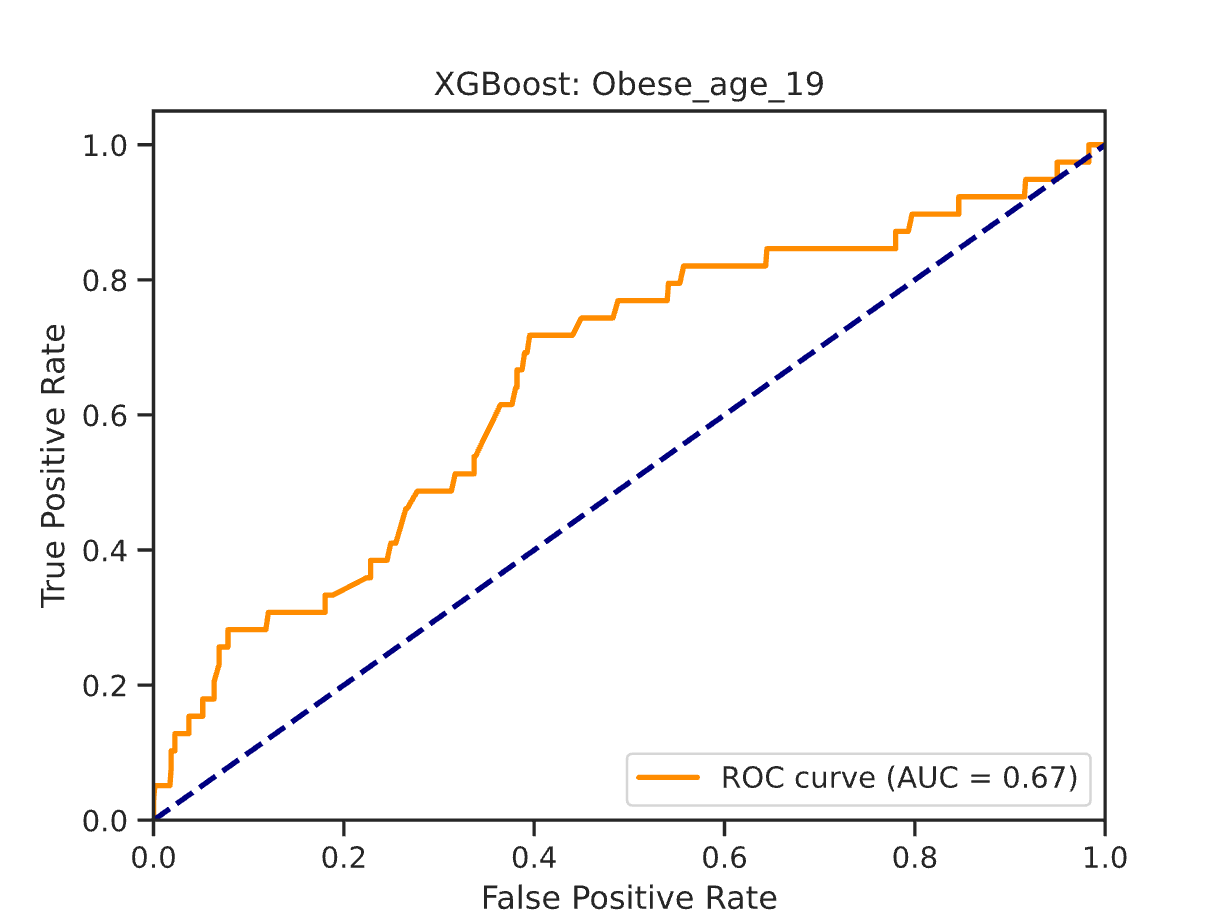**  **C**  **B** |
| --- |

**A**

**Supplementary Figure 2. Machine learning prediction of obesity using early-life factors and proteomics.** A-B) ML models predicting A) obesity classification (by IOTF standards) at age 19, using Extreme Gradient Boosting (XGBoost), and B) obesity diagnosis using the Histogram-based Gradient Boosting Classification Tree. C) Comparison of ML models predicting obesity diagnosis from selected proteomic markers in the cord blood.

| 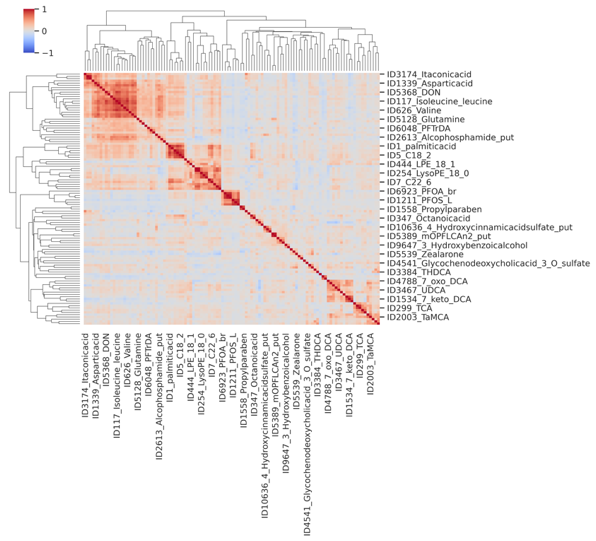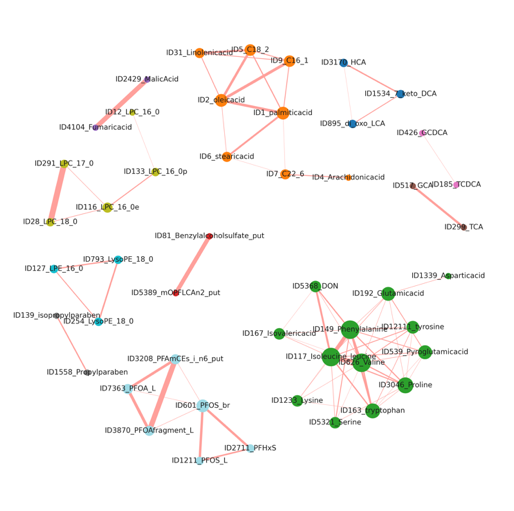  **B**  **A** |
| --- |

**Supplementary Figure 3. A-B: Clustering annotations for metabolites and environmental toxins, including their centroids.**

| 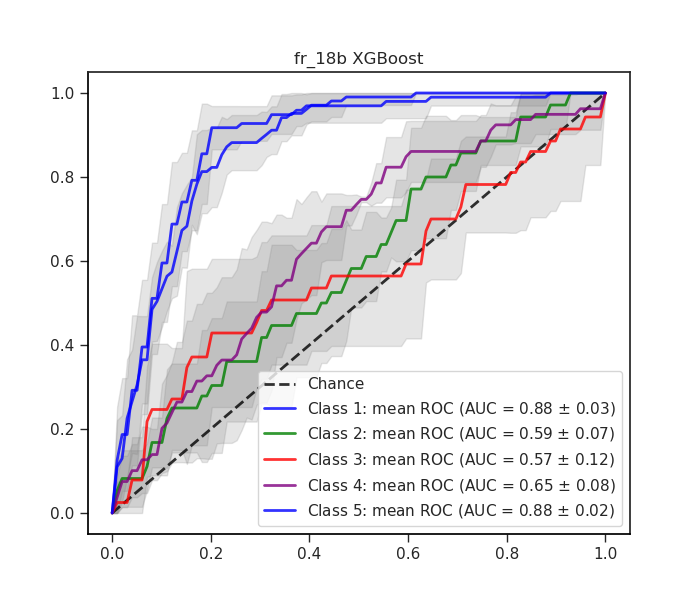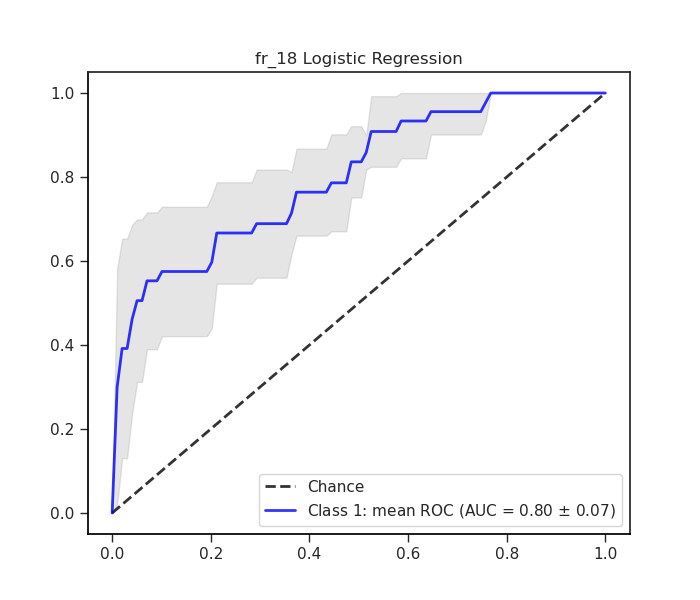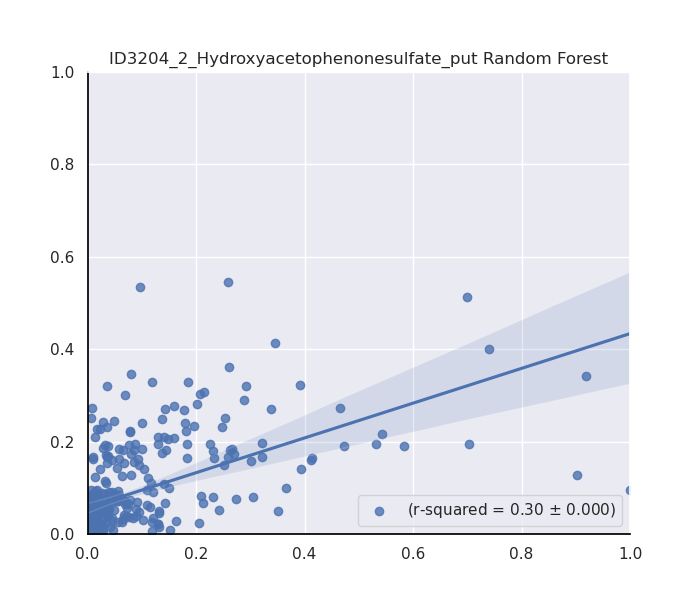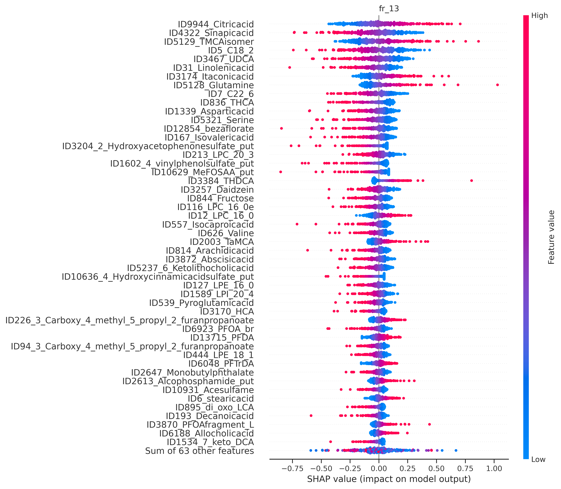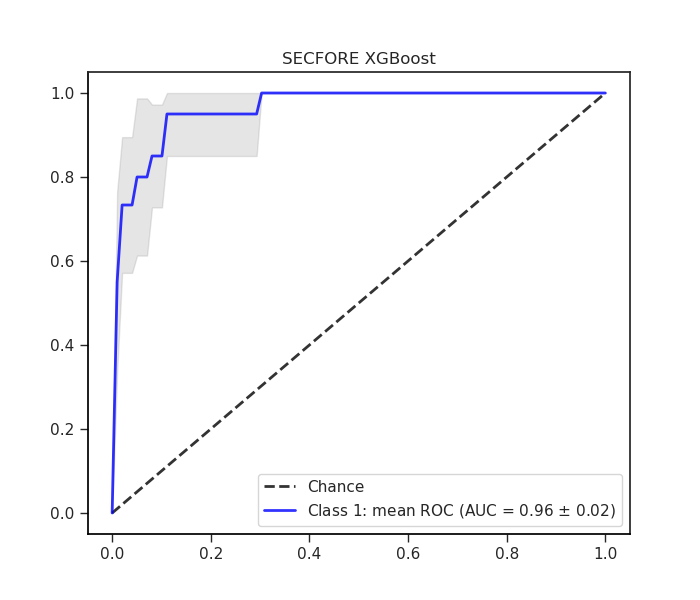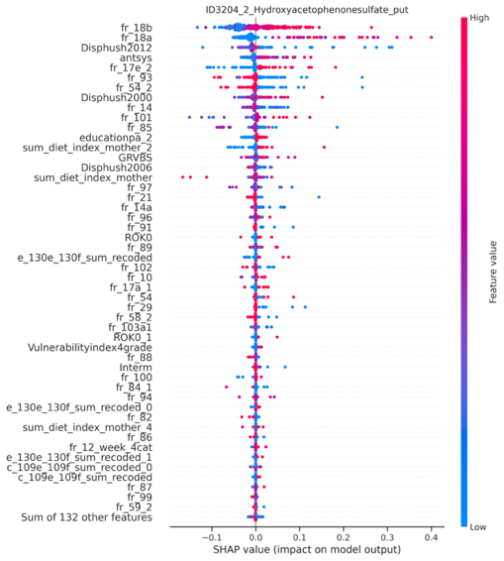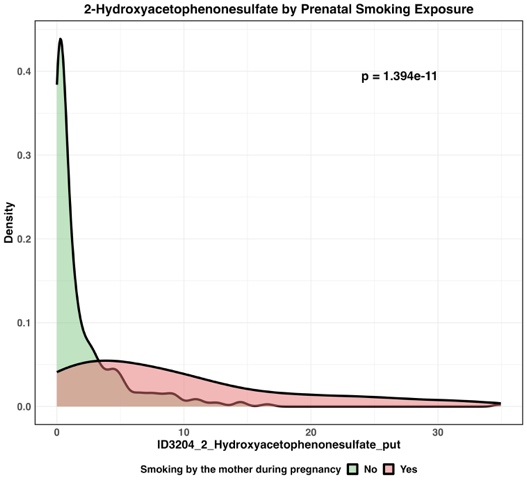  **Supplementary Figure 4. Influence of prenatal factors on the neonatal metabolome.**  **A**  **G**  **Fb**  **E**  **D**  **C**  **B** |
| --- |

A) Concentrations of 2-hydroxyacetophenonesulfate relative to maternal cigarette smoking during pregnancy.

B) Logistic regression analysis predicting maternal prenatal smoking exposure, displaying area under the curve

(AUC) statistics. C) Random Forest model predicting levels of 2-hydroxyacetophenonesulfate. D) Key drivers

of 2-hydroxyacetophenonesulfate concentrations in cord serum based on Shapley Additive Explanation

(SHAP) scores, illustrating the influence of each predictor. E) Prediction of maternal caffeine intake during

pregnancy (Class 1: never; 2: seldom; 3: sometimes; 4: often; 5: daily), with AUC statistics evaluated across

five folds. F) Significant metabolites linked to the mode of delivery. G) Extreme Gradient Boosting (XGBoost)

model illustrating the strong association between metabolites and C-section delivery.
